# Machine Learning for prediction of violent behaviors in schizophrenia spectrum disorders: a systematic review

**DOI:** 10.3389/fpsyt.2024.1384828

**Published:** 2024-03-21

**Authors:** Mohammadamin Parsaei, Alireza Arvin, Morvarid Taebi, Homa Seyedmirzaei, Giulia Cattarinussi, Fabio Sambataro, Alessandro Pigoni, Paolo Brambilla, Giuseppe Delvecchio

**Affiliations:** ^1^ Maternal, Fetal & Neonatal Research Center, Family Health Research Institute, Tehran University of Medical Sciences, Tehran, Iran; ^2^ Center for Orthopedic Trans-disciplinary Applied Research (COTAR), Tehran University of Medical Sciences, Tehran, Iran; ^3^ Sports Medicine Research Center, Neuroscience Institute, Tehran University of Medical Sciences, Tehran, Iran; ^4^ Department of Neuroscience (DNS), Padua Neuroscience Center, University of Padova, Padua, Italy; ^5^ Padua Neuroscience Center, University of Padova, Padua, Italy; ^6^ Department of Psychological Medicine, Institute of Psychiatry, Psychology and Neuroscience, Kings College London, London, United Kingdom; ^7^ Social and Affective Neuroscience Group, MoMiLab, Institutions, Markets, Technologies (IMT) School for Advanced Studies Lucca, Lucca, Italy; ^8^ Department of Pathophysiology and Transplantation, University of Milan, Milan, Italy; ^9^ Department of Neurosciences and Mental Health, Fondazione Istituto di Ricovero e Cura a Carattere Scientifico (IRCCS) Ca’ Granda Ospedale Maggiore Policlinico, Milan, Italy

**Keywords:** artificial intelligence, machine learning, schizophrenia, schizophrenia spectrum disorder, violent behavior

## Abstract

**Background:**

Schizophrenia spectrum disorders (SSD) can be associated with an increased risk of violent behavior (VB), which can harm patients, others, and properties. Prediction of VB could help reduce the SSD burden on patients and healthcare systems. Some recent studies have used machine learning (ML) algorithms to identify SSD patients at risk of VB. In this article, we aimed to review studies that used ML to predict VB in SSD patients and discuss the most successful ML methods and predictors of VB.

**Methods:**

We performed a systematic search in PubMed, Web of Sciences, Embase, and PsycINFO on September 30, 2023, to identify studies on the application of ML in predicting VB in SSD patients.

**Results:**

We included 18 studies with data from 11,733 patients diagnosed with SSD. Different ML models demonstrated mixed performance with an area under the receiver operating characteristic curve of 0.56-0.95 and an accuracy of 50.27-90.67% in predicting violence among SSD patients. Our comparative analysis demonstrated a superior performance for the gradient boosting model, compared to other ML models in predicting VB among SSD patients. Various sociodemographic, clinical, metabolic, and neuroimaging features were associated with VB, with age and olanzapine equivalent dose at the time of discharge being the most frequently identified factors.

**Conclusion:**

ML models demonstrated varied VB prediction performance in SSD patients, with gradient boosting outperforming. Further research is warranted for clinical applications of ML methods in this field.

## 1 Introduction

Schizophrenia disorders are characterized by delusions, hallucinations, disordered thinking, disorganized behavior, and blunted or inappropriate affects (1, 2). The disorders profoundly impact an individual’s quality of life and can also pose a risk to others, especially when they lead to violent behaviors (VB) (3). People with schizophrenia are frequently stigmatized as having a higher potential for violence, resulting in discrimination (4). Moreover, recent research has shown that schizophrenia spectrum disorders (SSD) – including schizophrenia, schizoaffective disorder, and other delusional disorders – have been linked with an increased risk of VB in various studies conducted worldwide (5–8).

The definition of VB is diverse, but it generally encompasses any manifestation of verbal or physical aggression directed at objects, others, or oneself (9, 10). The impact of VB is widespread, affecting not only the patients themselves, who may lose property, relationships, and well-being, but also their caregivers, such as family, friends, or healthcare workers, who can be traumatized by the experience (11, 12). Additionally, VB can increase the burden on the healthcare system for patients with SSD (13). A recent systematic review and meta-analysis reported a prevalence of 17.19 - 23.83% for different types of VB other than homicide among SSD patients (5). Another systematic review and meta-analysis, which pooled data from 15 countries, reported an odds ratio of 4.5 for interpersonal VB among SSD individuals compared to a general population group without these disorders (7).

Given the significant impact that VB can have on patients and those in their environment, it is critical to accurately predict the risk of VB to help prevent these behaviors. To date, many studies have investigated the risk factors for VB in SSD patients, including sociodemographic factors, disease characteristics, and previous patients’ medical history (14–16). However, most of these studies could not predict the risk of VB accurately, due to the complex and multifactorial nature of violence occurrence (17).

Machine learning (ML) is a subset of artificial intelligence that uses algorithms to learn from data, identify patterns, and make predictions (18, 19). By analyzing large amounts of data, ML algorithms can identify complex relationships and hidden links behind phenomena that are not obvious to human observers (20). The key aspect of ML is its capability to build predictive models, demonstrated by its ability to anticipate clinical outcomes such as suicidal ideation, impulsivity, and VB (19, 21, 22). This attribute renders ML a promising instrument for unraveling the intricate interplay between schizophrenia and VB, thereby aiding healthcare providers in the early identification of individuals susceptible to VB (23, 24). This, in turn, holds the potential to optimize resource allocation, diminish lay times, and fortify the safety of both staff and patients (25). Ultimately, the trajectory of ML in healthcare portends the evolution of medical prediction tools, envisaging their integration into routine clinical practice to proactively avert instances of VB and alleviate the burden of schizophrenia within this context (26).

This systematic review aims to investigate the potential of ML in predicting VB in patients with SSD, which we believe will offer a better understanding of the potential of ML in this clinical context and will be of interest to researchers and healthcare providers seeking to use ML to identify patients at risk of VB. Our main objectives are: 1) to discuss the most robust algorithms used for the prediction of VB; 2) to assess the general accuracy that has been achieved in predicting VB using ML; and 3) to review the effective factors that have enhanced ML’s ability to predict VB.

## 2 Materials and methods

### 2.1 Search strategy

We performed a systematic search in PubMed, Web of Sciences, Embase, and PsycINFO for relevant studies published before September 30, 2023. The search keywords consisted of three groups of keywords related to (a) ML, (b) SSD, and (c) VB. In this systematic review, the PICO (Population, Intervention, Comparison, Outcome) framework was employed with the following criteria:

Population: Schizophrenia spectrum disorder (SSD), including schizophrenia, schizoaffective disorder, and other delusional disorders (27);

Intervention: Machine learning models (ML);

Comparison: Medical records of patients or clinical violence risk assessment scales;

Outcome: Violent behavior (VB), defining as an attempt or action to harm a target, assault, robbery, aggression toward property, actions resulting in physical injury, child abuse, sexual abuse, threatening or causing injury with a weapon, verbal aggression or threatening, and violent crimes, e.g., attempted or completed homicide (28, 29).

This study was conducted in concordance with the Preferred Reporting Items for Systematic Reviews and Meta-Analysis (PRISMA) (30) and Critical Appraisal and Data Extraction for Systematic Reviews of Prediction Modelling Studies (CHARMS) guidelines (31).

### 2.2 Inclusion and exclusion criteria

All studies developing ML models for predicting VB in SSD patients were included. The development of a ML model in medicine includes the following stages: data acquisition, data preparation, ML model development, model evaluation, hyperparameter tuning, and model validation (32). We aimed to review the articles that developed and evaluated ML models for the prediction of VB in SSD patients. Hence, studies that only employed statistical models by using an ML subset (e.g., logistic regression) and did not either evaluate or validate the performance of their generated model were not included in this review. The exclusion criteria consisted of 1) Records that did not study patients with an SSD diagnosis, 2) records that did not predict VB, 3) records that did not employ an ML method, 4) records that were not available in the English language full-text, 5) editorials, commentaries, letters, conference abstracts, books, and review articles, and 6) animal studies.

### 2.3 Study selection

The selection process began with removing the duplicated records. Then, two authors (MP and AA) independently reviewed the article titles and abstracts and selected the relevant papers for the full-text screening process. The same authors (MP and AA) independently conducted the full-text screening of the selected records for eligibility. Any discrepancies were settled by discussion and, if necessary, referred to a third author (GC).

### 2.4 Data extraction

Two authors (AA and MT) conducted the data extraction. We collected data about the authors, year of publication, sample size, characteristics of the patients, ML model and validation techniques, input variables (i.e., demographics), output variables (VB), additional assessments, and key findings from every included record. Also, reported measures of the area under the receiver operating characteristic curves (AUROC), balanced accuracy, predictive power, P-value, sensitivity, specificity, positive predictive values (PPV), and negative predictive values (NPV) were collected. Cross-validation performance was defined as a training dataset because it involved data “seen” by the machine, whereas “unseen” data from a held-out test set or external cohort was treated as validation.

### 2.5 Data synthesis

To bypass the limitations of meta-analyzing heterogeneous datasets, one author (MT) implemented a novel comparative approach, ranking each ML model’s performance within individual studies and then averaging ranks across studies to identify the best overall performing ML model.

### 2.6 Risk of bias assessment

To assess the risk of bias (ROB), we employed the Prediction Model Risk of Bias Assessment Tool (PROBAST) (33). It is a tool for assessing ROB and the applicability of diagnostic and prognostic prediction model studies. PROBAST evaluates 4 domains of participants, predictors, outcome, and analysis in the study by 20 signaling questions. signaling questions of the PROBAST checklist and its guidance notes for rating ROB and applicability are fully provided in PROBAST checklist section of the **Supplementary Material.** These questions facilitate structured judgment of ROB in the studies of predictive models. We used the explanation and elaboration document that describes the rationale for including each domain and signaling question and guides researchers to use them to assess the ROB and applicability concerns. Also, to assess the ROB in the studies that employed more than one ML model, we selected the ML model with the best performance (best AUROC or accuracy).

## 3 Results

### 3.1 Study selection

**Figure 1:**
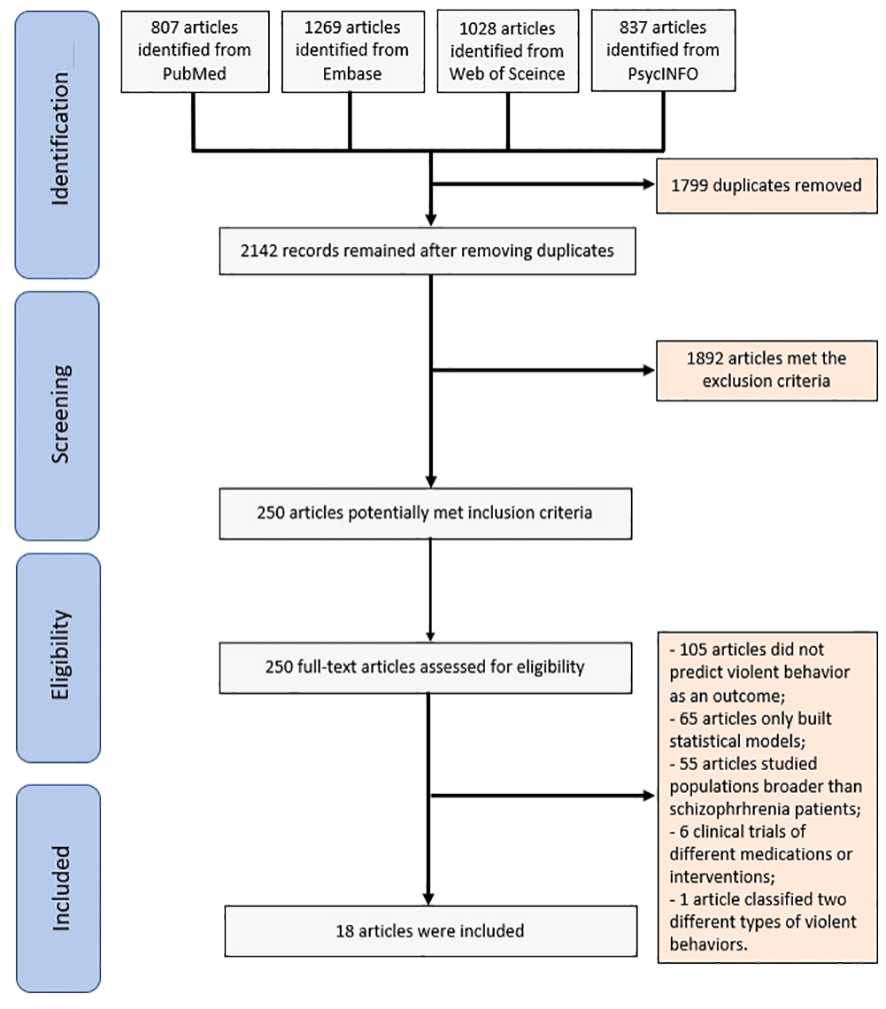
Study selection process flow diagram.

The search strategy employed in this systematic review yielded 3941 articles. Following the removal of duplicates, 2142 articles remained for further assessment. After assessing the abstracts, 250 articles were deemed suitable for full-text screening. A total of 18 articles satisfied the eligibility criteria and were included in the final analysis (**Figure 1**). **Table 1** shows the characteristics and extracted data of the included articles.

**Table 1:** Characteristics of the included studies.

Study	Diagnosis (Criteria)	Demographics	Trained features	Outcome variable	ML model	Accuracy	Other measures	Key Findings
(34) China	Schizophrenia (DSM-IV)	63 patients; - Violent/Non-violent: 48/15 - M/F: 38/25 - Mean age ± SD, y: 33.49 ± 10.42 - Single/Married: 54/9	9 sociodemographic-clinical features and 5 features of patient insight about their disease (total 14 features)	Violent behavior in one year (based on Chinese version of the VASA)	SVM (3-fold cross-validation)	Predictive Power: 76.2%		**Significant association with more violence tendency:** - ↓ age - male gender - ↓ insight 2 (ability to recognize and respond appropriately to early symptoms of relapse) - ↑ insight 3 (awareness and etiology-attribution of having schizophrenia) - ↑ insight 4 (Awareness of the achieved effect of treatment and likely compliance with treatment) **No association with violence tendency:** - Duration of illness - No. of hospital admissions - Education in years - Occupation - Marital status - Religion - Medication compliance - Insight 1 (awareness and description of psychotic symptoms) - Insight 5 (awareness of the social consequences of having schizophrenic disorders)
(35) China	SSD (DSM-IV)	107 patients; - M/F: 33/74 - Mean age ± SD, y: 33.4 ± 11.9 - Single/Married: 73/34	12 sociodemographic and baseline clinical features and 4 features of lipid profile (total 16 features)	The 18-item version of the Violence Scale in its Chinese translation (VS-C)	Stepwise LR	AUROC of all predictor variables (95% CI): 0.85 (0.72 - 0.97) Individual predictors: - Female gender: 0.64 (0.54 - 0.74) - AAO: 0.65 (0.51 - 0.78) - PSS: 0.71 (0.58 - 0.84) - NSS: 0.66 (0.51 - 0.80) - TC: 0.63 (0.50 - 0.75)		**Higher levels of violence:** - Significantly related to: - ↑ PSS - ↓ NSS - Not related to: - Female gender - Age - AAO - Education - Marital status - Employment status - Type of admission - BMI **↓ mean TC and TG levels** → show a trend with ↑ patients’ violence (p = 0.06). **Violence trajectories:** - Not associated with binary lipid profile: - TC - TG - LDL-C - HDL-C **↓ trajectory classes with an increasing level of violence** → ↑ TG levels
(36) China	Schizophrenia (DSM-IV)	77 patients [violent = 53], [non-violent = 23]; - M/F: [38/15], [13/11] - Mean age ± SD, y: [32.6 ± 4.7], [30.9 ± 5.2] - Single/Married: [47/6], [18/6]	20 sociodemographic features and 236 features of aminoacid, carbohydrate, and lipid metabolomics (total 256 features)	MacArthur Violence Risk Assessment Study (MVRAS)	RF + SVM (combination of them) (7-fold cross-validation)	AUROC of the biomarker panel formed by the three metabolites (ratio of L-asparagine to L-aspartic acid, vanillylmandelic acid, and glutaric acid) = 0.808		**The PCA →** no separation trend between the V.SCZ and NV.SCZ groups **Confirmed metabolic biomarkers of the V.SCZ group:** - D-ribose - 3-aminoisobutanoic acid - Glycerol 3-phosphate - Ratio of L-asparagine to L-aspartic acid - Glutaric acid - Ribitol - Vanillylmandelic acid - Glyceraldehyde - 3-aminosalicylic acid - 4-hydroxyproline **Plasma metabolites associated with V.SCZ diagnosed with SVM:** - Ratio of L-asparagine to L-aspartic acid - Vanillylmandelic acid - Glutaric acid **Metabolism pathways alteration in V.SCZ:** - glycerolipid metabolism pathway - Glycerol ↑ - Glycerol 3-phosphate ↓ - phenylalanine, tyrosine, and tryptophan biosynthesis pathway - 4-hydroxyphenyl pyruvic ↓
(37) Canada	SSD (DSM-IV)	275 patients [violent = 103], [non-violent = 172]; - M/F: [81/22], [116/56] - Mean age ± SD, y: [44.82 ± 12.95], [38.54 ± 12.83] - Single/Married: N/A	28 demographic, clinical, and sociocultural features	Retrospectively determined by patient electronic medical records for documentation of physically violent behavior (based on MOAS)	LR (stratified 5-fold cross validation) All values ± Standard Error	ACC: 0.57 ± 0.050 AUROC: 0.64 ± 0.066	SEN: 0.54 ± 0.065 SPE: 0.59 ± 0.044 PPV: 0.45 ± 0.057 NPV: 0.68 ± 0.044 P-Value: 0.013	**Significant association with more violence tendency:** - ↑ age - ↓ immigration after the age 18 - ↑ history of more than 5 times of hospitalizations - ↓ family history of mood disorders - ↓ NEO agreeableness - ↑ CTQ physical neglect **Borderline association with violence tendency:** - Male gender (p-value = 0.064) - ↓ NEO openness (p-value = 0.065) - ↑ CTQ physical abuse (p-value = 0.067) * No significant difference between seven classification models in a paired comparison of correct versus incorrect predictions. (based on McNemar’s test)
					LASSO (stratified 5-fold cross-validation) All values ± Standard Error	ACC: 0.60 ± 0.053 AUROC: 0.64 ± 0.065	SEN: 0.57 ± 0.062 SPE: 0.62 ± 0.055 PPV: 0.48 ± 0.061 NPV: 0.71 ± 0.045 P-Value: < 0.001	
					Elastic Net (stratified 5-fold cross validation) All values ± Standard Error	ACC: 0.59 ± 0.05 AUROC: 0.64 ± 0.06	SEN: 0.59 ± 0.059 SPE: 0.59 ± 0.041 PPV: 0.59 ± 0.047 NPV: 0.58 ± 0.042 P-Value: 0.003	
					RF (stratified 5-fold cross-validation) All values ± Standard Error	ACC: 0.62 ± 0.004 AUROC: 0.63 ± 0.005	SEN: 0.32 ± 0.008 SPE: 0.80 ± 0.004 PPV: 0.62 ± 0.008 NPV: 0.54 ± 0.003 P-Value: < 0.001	
					GB (stratified 5-fold cross-validation) All values ± Standard Error	ACC: 0.60 ± 0.037 AUROC: 0.63 ± 0.050	SEN: 0.22 ± 0.046 SPE: 0.82 ± 0.070 PPV: 0.49 ± 0.079 NPV: 0.64 ± 0.017 P-Value: 0.001	
					SVM (stratified 5-fold cross-validation) All values ± Standard Error	ACC: 0.57 ± 0.058 AUROC: 0.64 ± 0.067	SEN: 0.58 ± 0.082 SPE: 0.56 ± 0.049 PPV: 0.44 ± 0.062 NPV: 0.69 ± 0.055 P-Value: 0.026	
					RBF-SVM (stratified 5-fold cross-validation) All values ± Standard Error	ACC: 0.60 ± 0.046 AUROC: 0.62 ± 0.048	SEN: 0.45 ± 0.046 SPE: 0.69 ± 0.052 PPV: 0.48 ± 0.067 NPV: 0.67 ± 0.031 P-Value: < 0.001	
(38) China	Schizophrenia (ICD-10)	74 patients [violent = 42], [non-violent = 32]; - M/F: [42/0], [32/0] - Mean age ± SD, y: [30.88 ± 7.17], [28.00 ± 6.09] - Single/Married: [31/11], [25/7]	Sociodemographic - clinical features and three modalities neuroimaging data (T1-wieghted MRI, functional MRI and Diffusion tensor imaging) *LASSO used for feature selection	Violent offenders: those who had committed violent crimes, including killing or assaulting other people (interpersonal violence), and were undergoing forensic psychiatric evaluation (based on MOAS).	LASSO + SVM (combining the neuroimaging and sociodemographic-clinical features) (N/A-fold cross-validation)	ACC: 0.9067 AUROC: 0.95	SEN: 0.9091 SPE: 0.9048	**The V.SCZ is significantly associated with:** - Demographics: - ↓ lower education level - Clinical: (indicating ↑ severe psychotic symptoms in V.SCZ) - ↑ total BPRS score - ↑ BPRS hostility score - ↑ withdrawal factors - ↑ PCL-SV scale score - ↑ HCR-20 scale score **Significant overlapped regions that contributed to the prediction performance (shown by fMRI and sMRI):** - The cingulate gyrus - SFGdor - Temporal lobe (ITG and TP) - SMA - PAL **4 variables consisting model** (i.e., hostility-suspicion, psychopathy, the overall score of violence risk, and ↓ educational level) for distinguishing V.SCZ from NV.SCZ: - ACC: 0.80 - SEN: 0.7576 - SPE: 0.8333 - AUROC: 0.91 **The model of 3-way neuroimaging data fusion predicted violence:** - ACC: 0.8667 - SEN: 0.9394 - SPE: 0.8095 - AUROC: 0.91 T**he classifier model resulted of combining neuroimaging and sociodemographic-clinical features (outperforming model):** - ACC: 0.9067 - SEN: 0.9091 - SPE: 0.9048 - AUROC: 0.95
					LASSO + SVM (using sociodemographic & clinical variables) (N/A-fold cross-validation)	ACC: 0.80 AUROC: 0.91	SEN: 0.7576 SPE: 0.8333	
					LASSO + SVM (using Neuroimaging variables) (N/A-fold cross-validation)	ACC: 0.8667 AUROC: 0.91	SEN: 0.9394 SPE: 0.8095	
(39) Switzerland	SSD (ICD-9 & ICD-10) offenders: - Schizophrenia: 293 [violent = 241], [non-violent = 52]; - Schizoaffective disorder: 26 [violent = 21], [non-violent = 4]; - Acute psychotic disorder: 28 [violent = 17], [non-violent = 11];	369 patients [violent = 294], [non-violent = 75]; - M/F: [270/24], [68/7] - Mean age ± SD, y: [34.1 ± 10,4], [34.1 ± 9.6] - Single/Married: [233/56], [63/11] * Train to test ratio: 7:3	519 clinical and sociodemographic features *RF used for feature selection (10 features)	Violent offences (based on authors definition and Swiss law): homicide and attempted homicide, assault, rape, robbery, arson, and child abuse. Non-violent offenses: threat, theft, damage to property, minor sexual offenses (e.g., exhibitionism), drug offenses, illegal gun possession, and other minor offenses (e.g., triggering false alarms or emergency breaks)	GB (outperformed all the other ML algorithms) (5-fold cross validation)	With 95% CI: ACC: 0.6783 (0.6058, 0.7425) AUROC: 0.764 (0.6940, 0.8340)	With 95% CI: SEN: 0.7273 (0.6202, 0.8142) SPE: 0.6292 (0.5298, 0.7274) PPV: 0.6598 (0.5558, 0.7510) NPV: 0.7000 (0.5858, 0.7948)	**Most indicative factors for the distinction between V/NV offenses:** - The time spent in current forensic hospitalization - the age of first diagnosis of SSD - other influencing factors: - Time spent in prison - Olanzapine equivalent at discharge - PANSS total score at admission - PANSS total score at discharge - Previous convictions - Actual or potential discharge - Social isolation in adulthood - Poverty in childhood/adolescence **substance abuse →** was not found as a differentiating factor
					RF (5-fold cross validation)	ACC: 0.5819 AUROC: 0.7234	SEN: 0.2941 SPE: 0.8696 PPV: 0.3571 NPV: 0.8333	
					KNN (5-fold cross validation)	ACC: 0.5930 AUROC: 0.6693	SEN: 0.4902 SPE: 0.6957 PPV: 0.2841 NPV: 0.8471	
					LR (5-fold cross validation)	ACC: 0.6838 AUROC: 0.6787	SEN: 0.6863 SPE: 0.6812 PPV: 0.3465 NPV: 0.8998	
					Trees (5-fold cross validation)	ACC: 0.6097 AUROC: 0.6259	SEN: 0.4706 SPE: 0.7488 PPV: 0.3158 NPV: 0.8516	
					SVM (5-fold cross validation)	ACC: 0.6685 AUROC: 0.7223	SEN: 0.5882 SPE: 0.7488 PPV: 0.3659 NPV: 0.8807	
					NB (5-fold cross validation)	ACC: 0.6863 AUROC: 0.7576	SEN: 0.7059 SPE: 0.6667 PPV: 0.3429 NPV: 0.9020	
(40) China	Schizophrenia (DSM-III-R, DSM-IV or ICD-10)	**Discovery set:** 4711 patients [violent = 149], [non-violent = 4562]; - M/F: [113/36], [2617/1945] - Mean age: N/A - Single/Married: N/A **Validation set:** 3000 patients [violent = 85], [non-violent = 2915]; - M/F: [67/18], [1694/1221] - Mean Age: N/A - Single/Married: N/A	5 sociodemographic features and 76 features of psychotic symptoms (81 features)	History of interpersonal violence regardless of severity or the resulting injury.	Stepwise LR Model	**Discovery set:** - AUROC: 0.887 **Validation set:** - AUROC: 0.824		**Significant association with more violence tendency:** (After controlling for sociodemographic variables) - ↑ destruction of property - ↑ verbal aggression - ↓ delusion of persecution - ↓ flat affect - ↓ auditory hallucination - ↓ vagueness of thought: unstructured forms of thought (positive) - ↑ insomnia - ↓ poverty of thought: unstructured forms of thought (negative) * Delusions: different subtypes of delusions may have different effects on violent behavior. * Insomnia: severe insomnia is a prodromal sign of clinical exacerbation or relapse of schizophrenia * Medication subtypes: no contribution to violent behavior
(41) Switzerland	SSD (ICD-9 & ICD-10)	352 patients [violent = 113], [non-violent = 239]; - M/F: [108/5], [219/20] - Mean age ± SD, y: [32.64 ± 10.52], [34.62 ± 10.01] - Single/Married: [97/16], [118/233] * Train to test ratio: 7:3	508 features of sociodemographic data, childhood/youth experiences, psychiatric history, past criminal history, social/sexual functioning, details on the offense leading to forensic hospitalization, prison data, and particularities of the current hospitalization and psychopathological symptoms *RF used for feature selection (10 features)	Acts of aggression: either verbal or physical attacks aimed toward staff or other patients, as well as damage of property.	SVM (5-fold cross validation), (outperformed all the other ML algorithms)	with 95% CI: ACC: 0.735 (0.644 – 0.821) AUROC: 0.84 (0.75 – 0.93)	SEN: 0.835 (0.833 –0.838) SPE: 0.594 (0.588 –0.599) PPV: 0.835 (0.832 –0.838) NPV: 0.594 (0.588–0.599)	**Indicative factors in distinguishing aggressive and non-aggressive patients:** (Respectively) - Negative behavior toward other patients (the most indicative factor) - Breaking of ward rules - The PANSS score at admission - Poor impulse control (according to the PANSS) - Hostility (according to the PANSS) - Other influencing factors: - Complaints about hospital staff - Dis/antisocial utterances or attitudes - Tension - Uncooperativeness T**he most predictive variables regarding inpatient aggression:** - Psychopathology - Antisocial behavior * Severity of disease + interplay of the various factors → **development of aggression** (not only disease severity)
					RF (5-fold cross validation)	ACC: 0.753 AUROC: 0.83	SEN: 0.749 SPE: 0.749 PPV: 0.873 NPV: 0.599	
					GB (5-fold cross validation)	NA	NA	
					KNN (5-fold cross validation)	ACC: 0.777 AUROC: 0.85	SEN: 0.786 SPE: 0.768 PPV: 0.880 NPV: 0.631	
					LR (5-fold cross validation)	ACC: 0.749 AUROC: 0.85	SEN: 0.778 SPE: 0.721 PPV: 0.857 NPV: 0.606	
					Trees (5-fold cross validation)	ACC: 0.747 AUROC: 0.80	SEN: 0.726 SPE: 0.768 PPV: 0.862 NPV: 0.567	
					NB (5-fold cross validation)	ACC: 0.759 AUROC: 0.85	SEN: 0.879 SPE: 0.761 PPV: 0.878 NPV: 0.598	
(42) Switzerland	SSD offenders Subtypes: - Schizophrenia: 291 - Schizoaffective disorder: 27 - Other schizophrenic diagnoses: 52	369 patients; - Violent/Non-violent: 294/75 - M/F: 339/30 - Mean age ± SD, y: 34.1 ± 10,2 - Single/Married: 299/70	22 features of number and types of past stressors *Boosted tree used for feature selection	Violent offenses based on Swiss law: homicide and attempted homicide, assault, rape, robbery, arson, and child abuse. Nonviolent offenses: threat, theft, damage to property, minor sexual offenses (e.g., exhibitionism), drug offenses, illegal gun possession, and other minor offenses (e.g., triggering false alarms or emergency brakes).	Boosted classification trees (5-fold cross validation), (outperformed all the other ML algorithms)	ACC: 0.764 AUROC: 0.83	SEN: 0.8049 SPE: 0.7119 PPV: 0.66 NPV: 0.84	**A higher number** of past stressors → ↑ committing a violent offense **Boosted Classification Trees → outperforming model:** - ACC = 77% (LR ACC = 56.6%) **Model Performance With Stepwise Addition of Variables by Importance:** - N=1: AUROC: 0.47 - N=2: AUROC: 0.52 - N=3: AUROC: 0.57 - N=4: AUROC: 0.64 - N=5: AUROC: 0.76 - N=6-8: AUROC: 0.77 **The most important predictor of non-violent offense:** **- S**ocial isolation in adulthood - In violent offenses = 68% - In non-violent offenses = 84.9% **Factors associated with committing:** - Violent offenses: - Coercive psychiatric treatment in the past - Unemployment at the time of the index offense - Separation from main caregivers in childhood and/or youth - Non-violent offenses: - Social isolation **Failure in school →** not lead to ↑ committing a violent offense
					LR (5-fold cross validation)			
					SVM (5-fold cross validation)			
					KNN (5-fold cross validation)			
(43) China	Schizophrenia (DSM-V)	397 patients [violent = 146], [non-violent = 251]; - M/F: 397/0 - Mean age ± SD, y: [37.63 ± 12.56], [41.16 ± 14.62] - Single/Married: [122/24], [202/49] * Train to test ratio: 7:3	73 demographic, clinical, and sociocultural features *LASSO and LR used for feature selection (9 features)	Physical aggression against a person within 1 month before admission to the hospital	GLM (10-fold cross validation)	Balanced ACC: 0.5027 AUROC: 0.6454 (0.5327–0.7581)	Kappa: 0.0061 SEN: 0.1667 SPE: 0.8387	**Significant association with more violence tendency:** (determined by LASSO and LR) - ↓ education level - ↓ suicidal ideation (not consistent with previous studies’ findings) - ↑ cigarette smoking - ↑ positive syndrome - ↑ SDSS score **ML models result:** (using 5 factors determined above) - Best AUROC: NNET (AUROC = 0.6673) - Best ACC: KNN (ACC = 0.7352) - Best SEN: KNN (SEN = 0.5833)
					Rpart (10-fold cross validation)	Balanced ACC: 0.5757 AUROC: 0.6351 (0.5351–0.7350)	Kappa: 0.1608 SEN: 0.3611 SPE: 0.7903	
					NNET (10-fold cross validation)	Balanced ACC: 0.6416 AUROC: 0.6673 (0.5599–0.7748)	Kappa: 0.3007 SEN: 0.4444 SPE: 0.8387	
					KNN (10-fold cross validation)	Balanced ACC: 0.7352 AUROC: 0.5661 (0.4436–0.6886)	Kappa: 0.4934 SEN: 0.5833 SPE: 0.8871	
					RF (10-fold cross validation)	Balanced ACC: 0.7155 AUROC: 0.6353 (0.5218–0.7488)	Kappa: 0.4605 SEN: 0.5278 SPE: 0.9032	
					GLMNET (10-fold cross validation)	Balanced ACC: 0.5188 AUROC: 0.6449 (0.5323–0.7576)	Kappa: 0.0432 SEN: 0.1667 SPE: 0.8710	
					SVM (10-fold cross validation)	Balanced ACC: 0.5336 AUROC: 0.6400 (0.5223–0.7578)	Kappa: 0.0826 SEN: 0.0833 SPE: 0.9839	
					NB (10-fold cross validation)	Balanced ACC: 0.5963 AUROC: 0.6288 (0.5143–0.7433)	Kappa: 0.2152 SEN: 0.3056 SPE: 0.8871	
(44) China	Schizophrenia (ICD-10)	57 patients [Violent = 30], [Non-violent = 27]; - M/F: 57/0 - Mean age ± SD, y: [39.83 ± 11.03], [29.70 ± 8.87] - Single/Married: [11/19], [7/20] * Train to test ratio: 1:1	Sociodemographic-clinical features and structural magnetic resonance imaging (sMRI)	MOAS weighted total score ≥ 5 based on patients’ given history.	SVM (10-fold cross validation)	Balanced ACC: 0.8231 AUROC: 0.8410(0.6826–0.9995)	Kappa: 0.6429 SEN: 0.8000 SPE: 0.8462	**Significant association with more violence tendency:** - ↑ age - ↓ whole volume **Significant association with more violence tendency:** (After controlling for the whole-brain gray matter volume and age using the general linear model) - ↓ right bankssts thickness - ↓ right inferior parietal thickness - ↓ left frontalpole volume **ML models result:** (using 3 MRI features determined above) - Best AUROC: SVM (AUROC = 0.8410) - Best ACC: SVM (ACC = 0.8231) - Best SEN: RF and PDA (SEN = 0.8667) - Optimal model: SVM
					GLMNET (10-fold cross validation)	Balanced ACC: 0.7462 AUROC: 0.7692(0.5841–0.9544)	Kappa: 0.4948 SEN: 0.8000 SPE: 0.6923	
					Rpart (10-fold cross validation)	Balanced ACC: 0.7179 AUROC: 0.7179(0.5463–0.8896)	Kappa: 0.4315 SEN: 0.6667 SPE: 0.7692	
					RF (10-fold cross validation)	Balanced ACC: 0.7410 AUROC: 0.8077(0.6384–0.9770)	Kappa: 0.4896 SEN: 0.8667 SPE: 0.6154	
					PDA (10-fold cross validation)	Balanced ACC: 0.8179 AUROC: 0.8410(0.6760–1.000)	Kappa: 0.6392 SEN: 0.8667 SPE: 0.7692	
					KNN (10-fold cross validation)	Balanced ACC: 0.6795 AUROC: 0.6923(0.4882–0.8964)	Kappa: 0.3571 SEN: 0.6667 SPE: 0.6923	
					NNET (10-fold cross validation)	Balanced ACC: 0.7564 AUROC: 0.7641(0.5781–0.9501)	Kappa: 0.5051 SEN: 0.6667 SPE: 0.8462	
(45) Switzerland	offenders with SSD (ICD-9 or ICD-10) Subtypes: - Schizophrenia: 291 - Schizoaffective disorder: 27 - Delusional disorder: 52	370 patients; - M/F: 339/31 - Mean age ± SD, y: 34.1 ± 10.2 - Single/Married: 299/71 * Train to test ratio: 7:3	519 clinical and sociodemographic features *RF used for feature selection (10 features)	**First analysis:** CHC vs AOO including violent and non-violent offenses) **Second analysis:** committed homicide vs OVO based on Swiss law: attempted homicide, assault, rape, robbery, arson, and child abuse). * NVO: threat, theft, damage to property, minor sexual offenses (e.g., exhibitionism), drug offenses, illegal gun possession, and other minor offenses (e.g., triggering false alarms or emergency brakes)	LR (5-fold cross validation) CHC vs. AOO – CHC vs. VO	Balanced ACC: 0.6879 – 0.7071 AUROC: 0.7189 – 0.7973	SEN: 0.6649 – 0.7498 SPE: 0.6109 – 0.6643 PPV: 0.9311 – 0.9445 NPV: 0.23.56 – 0.2850	Aim 1: to identify the factors that distinguish between homicide/manslaughter and all other offenses committed by individuals with SSD. Aim 2: to identify the factors that distinguish between completed homicide/manslaughter and other violent (non-fatal) crimes. **Indicative variables in favor of homicide:** (for aim 1) - ↑ time spent in current forensic hospitalization - ↑ age at schizophrenia spectrum disorder diagnosis - ↑ time spent in prison > 1 year - ↓ daily cumulative olanzapine equivalent at discharge - ↑ age of patient at offence - ↓ PANSS Score at admission - ↓ future legal prognosis - ↑ age at admission - ↓ drug abuse in patients’ history - ↑ age of first inpatient treatment **Indicative variables in favor of homicide:** (for aim 2) - ↑ time spent in current forensic hospitalization - ↑ age at schizophrenia spectrum disorder diagnosis - ↑ time spent in prison > 1 year - ↓ daily cumulative olanzapine equivalent at discharge - ↓ amount of offenses leading to current forensic hospitalization - ↓ PANSS Score at admission - ↑ being forced for treatment - ↑ age at admission - ↓ no drug abuse in patients’ history - ↓ having legal complaints before current offense **Homicidal patients:** - ↓ previous criminal records (suddenly committing a very serious crime without any criminal history) - ↓ PANNS scores at admission - ↓ medication upon discharge - ↓ drug abuse (suggesting ↓ impaired brain structures) **ML models result:** (aim 1) - Best AUROC: GB (AUROC = 0.8249) - Best ACC: GB (ACC = 78.23) - Best SEN: RF (SEN = 95.34) **ML models result:** (aim 2) - Best AUROC: GB (AUROC = 0.8257) - Best ACC: GB (ACC = 77.45) - Best SEN: RF (SEN = 95.34) **GB model performance in the test dataset:** - AUROC: aim 1: 0.6672/aim2: 0.6912 - ACC: aim 1: 62.17/aim2: 70.21 - SEN: aim 1: 85.87/aim2: 78.87
					Tree (5-fold cross validation) CHC vs. AOO – CHC vs. OVO	Balanced ACC: 0.7351 – 0.5771 AUROC: 0.7591 – 0.6174	SEN: 0.8552 – 0.7504 SPE: 0.6178 – 0.4038 PPV: 0.9352 – 0.8949 NPV: 0.3208 – 0.2048	
					RF (5-fold cross validation) CHC vs. AOO – CHC vs. OVO	Balanced ACC: 0.5986 – 0.6468 AUROC: 0.7799 – 0.7455	SEN: 0.9534 – 0.9534 SPE: 0.2438 – 0.3422 PPV: 0.9086 – 0.8961 NPV: 0.280 – 0.5368	
					GB (5-fold cross validation) CHC vs. AOO – CHC vs. OVO	Balanced ACC: 0.7823 – 0.7745 AUROC: 0.8249 – 0.8257	SEN: 0.8379 – 0.8433 SPE: 0.6867 – 0.7057 PPV: 0.9529 – 0.9338 NPV: 0.3623 – 0.3967	
					KNN (5-fold cross validation) CHC vs. AOO – CHC vs. OVO	Balanced ACC: 0.6689 – 0.7563 AUROC: 0.8021 – 0.8254	SEN: 0.8755 – 0.8103 SPE: 0.4624 – 0.7022 PPV: 0.9279 – 0.9264 NPV: 0.3029 – 0.3267	
					SVM (5-fold cross validation) CHC vs. AOO – CHC vs. OVO	Balanced ACC: 0.6164 – 0.6537 AUROC: 0.7450 – 0.7703	SEN: 0.8795 – 0.8357 SPE: 0.3533 – 0.4717 PPV: 0.9134 – 0.9079 NPV: 0.2621 – 0.3695	
					NB (5-fold cross validation) CHC vs. AOO – CHC vs. OVO	Balanced ACC: 0.6945 – 0.6986 AUROC: 0.8023 – 0.8136	SEN: 0.7513 – 0.7272 SPE: 0.6378 – 0.6700 PPV: 0.9356 – 0.9421 NPV: 0.2311 – 0.2862	
					Final GB model evaluation in the test dataset CHC vs. AOO – CHC vs. OVO	Balanced ACC: 0.6217 – 0.7021 AUROC: 0.6672 – 0.6912	SEN: 0.8587 – 0.7887 SPE: 0.3846 – 0.6154 PPV: 0.5524 – 0.6512 NPV: 0.7547 – 0.7619	
(46) Switzerland	Offenders and non-offenders with SSD (ICD-10)	384 patients [offenders = 206], [non-offenders = 178]; - M/F: [187/19], [161/17] - Mean age ± SD, y: [34.8 ± 10.5], [35.4 ± 11.2] - Single/Married: N/A * Train to test ratio: 7:3	24 Sociodemographic, clinical and psychopharmacotherapy features	Offenses included both violent crimes—(attempted) homicide, assault, violent offenses against sexual integrity, robbery, and arson —and/or non-violent crimes —threat and coercion, property crime without violence, criminal damage, traffic offenses, drug offenses, and illegal gun possession.	LR (5-fold cross validation)	AUROC: 0.840 Balanced ACC: 0.7490	SEN: 0.6890 SPE: 0.8110 PPV: 0.7630 NPV: 0.7500	**ML models result:** - Best AUROC: SVM (AUROC = 0.870) - Best balanced ACC: SVM (balanced ACC = 0.778) - Best SEN: SVM (SEN = 0.78) - Best SPE: GB (SPE = 0.826) **8 Most important offense indicative factors ranked by SVM, respectively:** - ↑ olanzapine equivalent at time of discharge - ↓ regular intake of antipsychotic medication - ↓ additional antidepressant prescribed - ↑ olanzapine equivalent at time of admission - ↓ additional benzodiazepine prescribed - ↓ any antipsychotic medication in past - ↓ any outpatient treatment in the past - ↓ any inpatient treatment in the past **Other significant differences between offenders and non-offenders:** - Migration background - Education - Comorbid alcohol and substance abuse - Polypharmacy at admission No difference between offenders and non-offenders considering psychological severity (PANSS).
					Tree (5-fold cross validation)	AUROC: 0.790 Balanced ACC: 0.7390	SEN: 0.7090 SPE: 0.7710 PPV: 0.7260 NPV: 0.7550	
					RF (5-fold cross validation)	AUROC: 0.840 Balanced ACC: 0.7530	SEN: 0.7070 SPE: 0.7990 PPV: 0.7530 NPV: 0.7650	
					GB (5-fold cross validation)	AUROC: 0.850 Balanced ACC: 0.7620	SEN: 0.6980 SPE: 0.8260 PPV: 0.7820 NPV: 0.7600	
					KNN (5-fold cross validation)	AUROC: 0.820 Balanced ACC: 0.7460	SEN: 0.7090 SPE: 0.7820 PPV: 0.7410 NPV: 0.7570	
					SVM (5-fold cross validation) (outperformed all the other ML algorithms)	AUROC: 0.870 Balanced ACC: 0.7780	SEN: 0.7780 SPE: 0.7790 PPV: 0.7420 NPV: 0.7930	
					NB (5-fold cross validation)	AUROC: 0.850 Balanced ACC: 0.7700	SEN: 0.7650 SPE: 0.7760 PPV: 0.7450 NPV: 0.7950	
(47) China	Schizophrenia (ICD-10)	2037 patients [Aggressive = 611], [Non-aggressive = 1426]; - M/F: [401/210], [746/680] - Mean Age ± SD, y: [35.37 ± 10.29], [35.66 ± 10.62] - Single/Married: N/A * Train to test ratio: 7:3	19 demographic, clinical, and sociocultural features (ITAQ, Family APGAR, SSRS and FBS questionnaires score)	MOAS was applied to evaluate patients’ aggressive behaviors before they were discharged from the hospital, and a weighted total score of 4 or more was used as the inclusion criterion for the “group with significant aggressive behavior.”	RF (4-fold cross validation) (outperformed all the other ML algorithms)	AUROC: 0.955 (0.935 – 0.970) ACC: 0.889	SEN: 0.892 SPE: 0.887	**ML models result:** - Best AUROC: RF (AUROC = 0.955) - Best ACC: RF (ACC = 0.889) - Best SEN: SVM (SEN = 0.949) - Best SPE: RF (SPE = 0.887) **Top 8 features for predicting aggression based on RF model:** - ↑ APGAR score - ↑ ITAQ score - ↓ disease duration - ↑ history of attacks - ↓ SSRS score - ↓ medication adherence - ↑ age - ↓ FBS score
					SVM (4-fold cross validation)	AUROC: 0.902 (0.876 – 0.924) ACC: 0.827	SEN: 0.949 SPE: 0.770	
					MLP (4-fold cross validation)	AUROC: 0.904 (0.877 – 0.926) ACC: 0.866	SEN: 0.908 SPE: 0.847	
					LASSO (4-fold cross validation)	AUROC: 0.901 (0.874 – 0.923) ACC: 0.866	SEN: 0.908 SPE: 0.847	
(48) Switzerland	Offenders and non-offenders with SSD (ICD-9 or ICD-10)	740 patients [offenders = 370], [non-offenders = 370]; - M/F: [339/31], [339/31] - Mean age ± SD, y: [34.2 ± 10.2], [36.2 ± 12.2] - Single/Married: [297/73], [282/88] * Train to test ratio: 7:3	69 features of sociodemographic, illness-related factors, psychopharmacotherapy, adverse events during the referenced hospitalization, childhood/youth, and physical illness	Offenses included both violent crimes—(attempted) homicide, assault, violent offenses against sexual integrity, robbery, and arson —and/or non-violent crimes —threat and coercion, property crime without violence, criminal damage, traffic offenses, drug offenses, and illegal gun possession.	LR (5-fold cross validation)	AUROC: 0.870 Balanced ACC: 0.7740	SEN: 0.7320 SPE: 0.8160 PPV: 0.7890 NPV: 0.7630	**ML models result:** - Best AUROC: GB (AUROC = 0.910) - Best balanced ACC: GB (balanced ACC = 0.811) - Best SEN: KNN (SEN = 0.842) - Best SPE: GB (SPE = 0.832) **8 Most important offense indicative factors ranked by GB, respectively:** - ↑ olanzapine equivalent at time of discharge - ↑ migration background - ↓ medication compliance - ↓ failures during temporary leave - ↓ outpatient treatment before referenced hospitalization - ↓ preexisting physical or neurological illness - ↑ no compulsory school graduation - ↓ inpatient treatment before referenced hospitalization Psychopathology and aggression related items itself did not show a heavy influence on the model.
					Tree (5-fold cross validation)	AUROC: 0.840 Balanced ACC: 0.8050	SEN: 0.8290 SPE: 0.7810 PPV: 0.7770 NPV: 0.8250	
					RF (5-fold cross validation)	AUROC: 0.890 Balanced ACC: 0.7740	SEN: 0.7370 SPE: 0.8110 PPV: 0.7870 NPV: 0.7630	
					GB (5-fold cross validation) (outperformed all the other ML algorithms)	AUROC: 0.910 Balanced ACC: 0.8110	SEN: 0.7900 SPE: 0.8320 PPV: 0.8180 NPV: 0.8100	
					KNN (5-fold cross validation)	AUROC: 0.850 Balanced ACC: 0.7980	SEN: 0.8420 SPE: 0.7550 PPV: 0.7630 NPV: 0.8350	
					SVM (5-fold cross validation)	AUROC: 0.880 Balanced ACC: 0.7820	SEN: 0.7480 SPE: 0.8160 PPV: 0.7960 NPV: 0.7760	
					NB (5-fold cross validation)	AUROC: 0.880 Balanced ACC: 0.7780	SEN: 0.8230 SPE: 0.7330 PPV: 0.7450 NPV: 0.8130	
(49) Switzerland	Offenders and non-offenders with SSD (ICD-9 or ICD-10)	740 patients [offenders = 370], [non-offenders = 370]; - M/F: [339/31], [339/31] - Mean age ± SD, y: [34.2 ± 10.2], [36.2 ± 12.2] - Single/Married: [297/73], [282/88] * Train to test ratio: 7:3	194 demographic and illness-related features	Offenses included both violent crimes—(attempted) homicide, assault, violent offenses against sexual integrity, robbery, and arson —and/or non-violent crimes —threat and coercion, property crime without violence, criminal damage, traffic offenses, drug offenses, and illegal gun possession.	LR (5-fold cross validation)	AUROC: 0.830 Balanced ACC: 0.7610	SEN: 0.7930 SPE: 0.7300 PPV: 0.7320 NPV: 0.7910	**ML models result:** - Best AUROC: GB (AUROC = 0.880) - Best balanced ACC: GB (balanced ACC = 0.7850) - Best SEN: KNN (SEN = 0.817) - Best SPE: GB (SPE = 0.804) **6 Most important offense indicative factors ranked by GB, respectively (all are related to treatment):** - ↑ olanzapine equivalent at time of discharge - ↓ history of medication compliance - ↑ compulsory measure during referenced hospitalization - ↓ outpatient treatment before referenced hospitalization - ↓ neuroleptic medication in psychiatric history - ↓ any other type of psychopharmacotherapy in the past **Important non-dominant factors in the model (related to the psychopathology):** - The severity of psychopathology - Type of symptoms - Comorbid substance use - Comorbid personality disorders
					Tree (5-fold cross validation)	AUROC: 0.830 Balanced ACC: 0.7610	SEN: 0.7280 SPE: 0.7940 PPV: 0.7720 NPV: 0.7560	
					RF (5-fold cross validation)	AUROC: 0.860 Balanced ACC: 0.7540	SEN: 0.7890 SPE: 0.7190 PPV: 0.7250 NPV: 0.7860	
					GB (5-fold cross validation) (outperformed all the other ML algorithms)	AUROC: 0.880 Balanced ACC: 0.7850	SEN: 0.7660 SPE: 0.8040 PPV: 0.7840 NPV: 0.7830	
					KNN (5-fold cross validation)	AUROC: 0.810 Balanced ACC: 0.7450	SEN: 0.8170 SPE: 0.6730 PPV: 0.7010 NPV: 0.8060	
					SVM (5-fold cross validation)	AUROC: 0.820 Balanced ACC: 0.7350	SEN: 0.7430 SPE: 0.7260 PPV: 0.7150 NPV: 0.7490	
					NB (5-fold cross validation)	AUROC: 0.830 Balanced ACC: 0.7590	SEN: 0.7840 SPE: 0.7340 PPV: 0.7330 NPV: 0.7840	
(50) Switzerland	Suicidal offenders and non-offenders with SSD (ICD-9 or ICD-10)	399 patients [offenders = 232], [non-offenders = 167]; - M/F: [211/21], [152/15] - Mean age ± SD, y: [33.5 ± 9.6], [35.7 ± 11.9 - Single/Married: [180/52], [123/44] * Train to test ratio: 7:3	107 Sociodemographic, clinical and psychopharmacotherapy features	Offenses included both violent crimes—(attempted) homicide, assault, violent offenses against sexual integrity, robbery, and arson —and/or non-violent crimes —threat and coercion, property crime without violence, criminal damage, traffic offenses, drug offenses, and illegal gun possession.	LR (5-fold cross validation)	AUROC: 0.757 Balanced ACC: 0.6250	SEN: 0.7930 SPE: 0.4550 PPV: 0.5040 NPV: 0.7690	**ML models result:** - Best AUROC: NB (AUROC = 0.870) - Best balanced ACC: GB (balanced ACC = 0.7690) - Best SEN: Tree (SEN = 0.852) - Best SPE: SVM (SPE = 0.932) **6 Most important offense indicative factors ranked by NB, respectively:** - ↓ antidepressant during referenced hospitalization - ↓ regular intake of antipsychotic medication - ↓ any outpatient treatment in the past - ↓ global cognitive deficit - ↓ PANSS: lack of spontaneity - ↓ PANSS: anxiety
					Tree (5-fold cross validation)	AUROC: 0.756 Balanced ACC: 0.6750	SEN: 0.8520 SPE: 0.4980 PPV: 0.5440 NPV: 0.8280	
					RF (5-fold cross validation)	AUROC: 0.757 Balanced ACC: 0.6620	SEN: 0.8400 SPE: 0.4830 PPV: 0.5290 NPV: 0.8220	
					GB (5-fold cross validation)	AUROC: 0.850 Balanced ACC: 0.7690	SEN: 0.6510 SPE: 0.8870 PPV: 0.7860 NPV: 0.7940	
					KNN (5-fold cross validation)	AUROC: 0.765 Balanced ACC: 0.6410	SEN: 0.8190 SPE: 0.4630 PPV: 0.5130 NPV: 0.8020	
					SVM (5-fold cross validation)	AUROC: 0.850 Balanced ACC: 0.7370	SEN: 0.5420 SPE: 0.9320 PPV: 0.8440 NPV: 0.7470	
					NB (5-fold cross validation) (outperformed all the other ML algorithms)	AUROC: 0.870 Balanced ACC: 0.7660	SEN: 0.6360 SPE: 0.8970 PPV: 0.8110 NPV: 0.7770	
(51) Canada	Schizophrenia (DSM-V)	196 patients [Aggressive = 26], [Non-aggressive = 170]; - M/F: [19/7], [149/21] - Mean age ± SD, y: [37.53 ± 11.87], [41.75 ± 13.12] - Single/Married: N/A * Train to test ratio: 6:4	67 demographic and clinical features (evidence-based risk factors, protective factors and factors related to the course of treatment) plus clinician judgement of violence	Binary classification was used to dichotomize physically aggressive (AIS ≥4) and non-physically aggressive incidents (AIS <3) at follow-up timepoints.	Boosted LR (cross validation (NA fold))	**At 4 months F/U:** AUROC: 0.903 (0.858 – 0.942) ACC: 0.7420 (0.7868 – 0.8871) Balanced ACC: 0.8416	**At 4 months F/U:** SEN: 0.6190 SPE: 0.8650 PPV: 0.3250 NPV: 0.9558	**ML models result:** **-** Best performance in predicting VB in 4 months: RF **-** Best performance in predicting VB in 12 months: RF **-** Best performance in predicting VB in 18 months: Extreme GB **Most important predictive features of VB across 4-, 12- and 18-months F/U:** - Change in attitude/cooperation - Change in rule adherence - Change in impulse control - Change in stress management - Worsening mood and psychotic symptoms - Change in family support - Presence of personality disorders - Peer influence RF model combining sociodemographic and clinical features with clinician-rated likelihood of violence showed superior performance comparing with sociodemographic and clinical features alone: - Balanced ACC: 0.9161 - SEN: 0.9523 - SPE: 0.8800 - PPV: 0.4545 - NPV: 0.9943
					Elastic Net (cross validation (NA fold))	**At 4 months F/U:** AUROC: 0.815 (0.656 – 0.947) ACC: 0.8326 (0.7767 – 0.8793) Balanced ACC: 0.8222	**At 4 months F/U:** SEN: 0.8095 SPE: 0.8350 PPV: 0.3400 NPV: 0.9766	
					LASSO (cross validation (NA fold))	**At 4 months F/U:** AUROC: 0.712 (0.584 – 0.833) ACC: 0.5973 (0.5294 – 0.6625) Balanced ACC: 0.6283	**At 4 months F/U:** SEN: 0.6666 SPE: 0.5900 PPV: 0.1458 NPV: 0.9440	
					KNN (cross validation (NA fold))	**At 4 months F/U:** AUROC: 0.669 (0.551 – 0.784) ACC: 0.7376 (0.6743 – 0.7943) Balanced ACC: 0.6419	**At 4 months F/U:** SEN: 0.5238 SPE: 0.7600 PPV: 0.1864 NPV: 0.9382	
					Adaptive Boosting (cross validation (NA fold))	**At 4 months F/U:** AUROC: 0.826 (0.764 – 0.883) ACC: 0.8145 (0.7569 – 0.8635) Balanced ACC: 0.7483	**At 4 months F/U:** SEN: 0.6666 SPE: 0.8300 PPV: 0.2916 NPV: 0.9595	
					Extreme GB (outperformed all the other ML algorithms at 18-months F/U) (cross validation (NA fold))	**At 4 months F/U:** AUROC: 0.928 (0.885 – 0.963) ACC: 0.8281 (0.7717 – 0.8754) Balanced ACC: 0.8625 **At 18 months F/U:** AUROC: 0.870 (0.814 – 0.918) ACC: 0.8343 (0.7719 – 0.8853) Balanced ACC: 0.8181	**At 4 months F/U:** SEN: 0.9047 SPE: 0.8200 PPV: 0.3454 NPV: 0.9879 **At 18 months F/U:** SEN: 0.8000 SPE: 0.8362	
					RF (outperformed all the other ML algorithms at 4 and 12-months F/U) (cross validation (NA fold))	**At 4 months F/U:** AUROC: 0.914 (0.872 – 0.951) ACC: 0.8733 (0.8221 – 0.9141) Balanced ACC: 0.8660 **At 12 months F/U:** ACC: 0.8010 (0.7373 – 0.8552)	**At 4 months F/U:** SEN: 0.8571 SPE: 0.8750 PPV: 0.4186 NPV: 0.9831 **At 12 months F/U:** SEN: 0.9333 SPE: 0.7897	
					Bagged CART (cross validation (NA fold))	**At 4 months F/U:** AUROC: 0.928 (0.886 – 0.964) ACC: 0.7647 (0.7032 – 0.8190) Balanced ACC: 0.7421	**At 4 months F/U:** SEN: 0.7142 SPE: 0.7700 PPV: 0.2459 NPV: 0.9650	
					Conditional Forrest (cross validation (NA fold))	**At 4 months F/U:** AUROC: 0.914 (0.869 – 0.953) ACC: 0.7738 (0.7128 – 0.8272) Balanced ACC: 0.8536	**At 4 months F/U:** SEN: 0.9523 SPE: 0.7550 PPV: 0.2898 NPV: 0.9934	

AA, Aminoacid; AAO, Age at onset; ACC, Accuracy; AIS, Aggressive Incidents Scale; AOO, All Other Offenses (excluding committed homicide); APGAR, Adaptation; Partnership; Growth; Affection; and Resolve; AUROC, Area Under the Receiver Operator Characteristic Curve; BIS-11, Barratt Impulsiveness Scale-11; BMI, Body Mass Index; BPRS, Brief Psychiatric Rating Scale; CA, Compulsory Admission; CART, Classification and Regression Trees; CI, Confidence Interval; CHC, Committed Homicide; CTQ, Childhood Trauma Questionnaire; F, Female; FBS, Family Burden Scale of Disease; F/U, Follow-up; GLM, Generalized Linear Model; GB, Gradient Boosting; GLMNET, Generalized Linear Model Neural Net; HCR-20: Historical; Clinical and Risk Management 20; HDL-C, High Density Lipoprotein Cholesterol; ITAQ, Insight and Treatment Attitude Questionnaire; ITG, Inferior Temporal Gyrus; KNN, K-Nearest Neighbor; LDL-C, Low Density Lipoprotein Cholesterol; LASSO, Least Absolute Shrinkage and Selection Operator; LR, Logistic Regression; M, Male; ML, machine learning; MLP, Multi-layer Perceptron; MOAS, Modified Overt Aggression Scale; NA: not available; NB: Naive Bayes; NEO, NEO-Five Factor Inventory including Neuroticism; Emotional stability; Extraversion; Openness to experience; Agreeableness; and Conscientiousness; NNET, Neural Net; NPV, Negative Predictive Value; NSS, Negative Symptoms Score; NVO, Non-Violent Offenses; NV.SCZ, Non-violent Schizophrenia; OVO, Other Violent Offenses; PAL, Pallidum; PANSS, Positive And Negative Symptom Scale; PCA, Principal Component Analysis; PCL-SV, Psychopathy Checklist-Screening Version; PDA, Penalized Discriminant Analysis; PPV, Positive Predictive Value; PSS, Positive Symptoms Score; RBF-SVM, SVM classifiers with Radial Basis Function kernels; RF, Random Forest; SD, Standard Deviation; SDSS: Social Disability Screening Schedule; measures a patient’s social; occupational; and psychological functioning; SEN, Sensitivity; SFGdor, dorsolateral part of Superior Frontal Gyrus; SIP, Schedule for Assessment of Insight in Psychosis; SMA, Supplementary Motor Area; SPE, Specificity; SSD, Schizophrenia-Spectrum Disorders; SSRS, Social Support Rating Scale; SVM, Support Vector Machine; TC, Total Cholesterol; TG, Triglyceride; TP, Temporal Pole; VA, Volunteer Admission; VASA, Violence and Suicide Assessment Scale; V.SCZ, Violent Schizophrenia.

### 3.2 Study characteristics

#### 3.2.1 General features

The 18 included studies were conducted in Switzerland (n=8), China (n=8), and Canada (n=2). A total of 11,733 patients diagnosed with SSD were systematically reviewed in the present study, with diagnostic criteria including Diagnostic and Statistical Manual of Mental Disorders (DSM)-III, IV, and V, International Classification of Diseases (ICD)-9 and 10. Of the patients, 7,330 (62.47%) were male, and 4,403 (37.53%) were female. Three studies included exclusively male participants (38, 43, 44). Except for one study that recruited outpatients (34), all other studies recruited participants from inpatient settings. Among these studies, four employed ML models to predict VB during the current admission (35, 41, 47, 51). Additionally, nine studies categorized patients based on the occurrence of VB prior to their current admission (38–40, 43, 44, 46, 48–50), while another four classified patients into violent and non-violent groups by retrospectively reviewing their medical records since their disease onset (36, 37, 42, 45). Moreover, eight studies were part of a larger project investigating the relationship between SSD and offending and used the same dataset of offender patients as their sample population (39, 41, 42, 45, 46, 48–50).

#### 3.2.2 Input measures

Most of the included studies utilized only sociodemographic and clinical features of patients to predict VB. Of these studies, five evaluated a large number of features (over 100 features) as predictors (39, 41, 45, 49, 50). Tzeng et al. (2004) explored the role of schizophrenia patients’ insight about their disease as a variable in addition to the sociodemographic features to predict the occurrence of VB (34). Additionally, Sun et al. (2021) explored the correlation between different psychotic symptoms and violence among schizophrenia patients (40). Likewise, Kirchebner et al. (2022) analyzed the role of accumulation and types of stressors in the patient’s history in increasing the severity of an offense (42). Furthermore, Machetanz et al. (2022, 2023) in two separate studies evaluated the differences between offender and non-offender SSD patients regarding psychiatric pr**e**scription patterns and illness-related factors (46, 49). Also, ten studies analyzed the relationship between different rating tools scores and VB in patients with SSD (36, 38, 39, 41, 43, 45, 46, 48–50), including the Brief Psychiatric Rating Scale (BPRS) (38, 43, 52), the Psychopathy Checklist: Screening Version (PCL-SV), the Historical, Clinical and Risk management (HCR-20) scale (38, 53), The Barratt Impulsiveness Scale version 11 (BIS-11) (38, 54), the Positive And Negative Symptom Scale (PANSS) (36, 39, 41, 43, 45, 46, 48–50, 55), the Social Disability Screening Schedule (SDSS) (43), Insight and Treatment Attitude Questionnaire (ITAQ) (47, 56), Family Adaptation, Partnership, Growth, Affection and Resolve (APGAR) (47, 57), Social Support Rating Scale (SSRS) (47, 58), and Family Burden Scale of Disease (FBS) (47, 59). Furthermore, two studies evaluated neuroimaging data of patients as VB predictors, along with sociodemographic features. Specifically, Gou et al. (2021) attempted to combine three modalities of neuroimaging data – T1-weighted magnetic resonance imaging (MRI), functional magnetic resonance imaging (fMRI), and diffusion tensor imaging (DTI) – with patients’ clinical features to improve the prediction power of the ML model (38). Similarly, Yu et al. (2022) assessed the effects of structural MRI (sMRI) features such as gray matter volume (GMV), cortical surface area, and cortical thickness in differentiating between violent and non-violent schizophrenia patients (44).

Moreover, two other studies examined the role of biochemical markers in indicating VB. Chen et al. (2015) examined the relationship between the violence trajectories, baseline clinical features, and lipid levels to develop a model to predict more violent trajectories (35), while Chen et al. (2020) tried to identify the metabolic characteristic of violent schizophrenia patients, including amino acids, lipids, and carbohydrates metabolism, by performing untargeted metabolomics and analyzing their plasma metabolites (36).

#### 3.2.3 Output measures

The definition of VB varied significantly across studies due to the use of different criteria, scales, or aims. While some studies defined verbal aggression as VB, others only included physical aggression, and some differentiated offenses based on their severity. Four studies utilized the Modified Overt Aggression Scale (MOAS) (60) criteria, but with different thresholds (37, 38, 44, 47): Wang et al. (2020) considered the outcome as physical aggression, irrespective of the aim or the outcome of VB (37), Gou et al. (2021) considered it as physical aggression aimed at others and leading to injury (38), and finally Yu et al. (2022) and Cheng et al. (2023) defined VB as a minimum MOAS score of 5 or 4 respectively, which could be achieved by various VBs without restricting the type or the target of it (44, 47). Additionally, four studies employed different scales for the VB definition: Tzeng et al. (34) used the Violence and Suicide Assessment (VAS-A) (61), Chen et al. (35) utilized the Violence Scale (28), Chen et al. (36) employed the MacArthur Violence Risk Assessment Study (MVRAS) (62), and Watts et al. (51) used the Aggressive Incidents Scale (AIS) (63). Meanwhile, three other studies simply defined VB without the use of any scale: Sun et al. (2021) and You et al. (2022) focused on physical VB aimed at others (40, 43), while Hoffman et al. (2022) included physical VB regardless of the aim (41). On the other hand, six studies used a shared database to distinguish between violent and non-violent offenses (39, 42, 46, 48–50). In a seventh study, they attempted to predict the risk of homicide among other offenses (45).

### 3.3 Machine learning

#### 3.3.1 Overview of algorithms

None of the 18 studies utilized unsupervised learning (clustering), which is consistent with the nature of the subject – since the classes and the target of classification is given (64). Instead, all of them used supervised learning (classification or regression), with three studies (43, 44, 47) incorporating deep learning through the neural network (NNET) or multi-layer perceptron (MLP) model. Among the top classification methods of supervised learning, support vector machine (SVM) was utilized in fifteen studies, decision trees (including random forests (RF) in fifteen, and k-nearest neighbor (KNN) in eleven. For the top regression methods of supervised learning, logistic regression (LR) (including stepwise LR) was utilized by twelve studies, while least absolute shrinkage and selection operator (LASSO) was used by five. While thirteen studies compared different ML models’ functions in violence prediction, others focused on developing a single prediction model (34–36, 38, 40). See **Supplementary Material** for detailed information regarding the model development and validation across the reviewed studies.

#### 3.3.2 Model development

In most of the studies, some details were unclear about model development, with few providing information about hyperparameter tuning, an essential part of model development. Hyperparameters are parameters set before the training process begins and affect how the model learns from and generalizes the data (65). Tuning hyperparameters can significantly impact model performance and determine the complexity/flexibility of the model (65). Among the eighteen studies, four provided some explanation about the hyperparameter tuning (34, 35, 38, 47), two used default settings without optimization (41, 45), and the other twelve studies did not mention anything about hyperparameter optimization.

One study did not develop a prediction model but sought to find the best predictors of violence in SSD by using SVM and LR separately (36). Then they identified overlapping best predictors among metabolic biomarkers. By using two different models separately, they aimed to minimize overfitting – a common bias where models fit too closely to the training data, producing good predictions for data points in the training set but do not generalize well to new data, performing poorly on new samples (65) – as it is unlikely for two different algorithms to overfit the same way.

The remaining studies developed and assessed models for violence prediction in SSD. They employed feature selection or cross-validation to overcome overfitting bias and achieve more accurate model development. Seven studies employed data-driven feature selection by ML before model training to control overfitting: one utilized LASSO (38), three used RF (39, 41, 45), one applied boosted tree (42), one utilized both LASSO and LR (43), and one selected features after calculation of variable importance for each employed model separately (51). Sixteen studies used cross-validation, with two using 10-fold cross-validation (43, 44), one using 7-fold (36), nine using 5-fold (37, 39, 41, 42, 45, 46, 48–50), one using 4-fold (47), and one using 3-fold (34). Two studies did not use cross-validation (35, 40).

Furthermore, only sixteen studies acknowledged the implementation of imputation methods on their respective training set data (39, 41, 45, 46, 49–51). Imputation methods refers to techniques for estimating or imputing missing values within datasets to enhance overall completeness and analytical suitability (66). Notably, 5 studies opted for a common practice wherein missing continuous values were imputed with the mean observed values pertaining to the respective variable, while categorical variables underwent replacement with the mode of observed values (39, 41, 45, 46, 49, 50). However, one study imputed missing continuous variables with either the observed mean or median values, concurrently addressing missing categorical variables based on the mode of observed values (51).

The choice of ML models is often influenced by the type of data being used. According to a survey (67), deep learning models, such as NNET and MLP, are commonly employed for interpreting imagery data. Among the studies we reviewed, two specifically utilized brain imaging data to train ML models: In one study, LASSO was employed for image interpretation, while SVM was used for integrating image and clinical data and making final predictions (38). In the other study, seven models, including NNET, were compared to assess their performance (44).

#### 3.3.3 Model validation

Regarding model validation and generalization assessment, six studies reported results on the training set (34–38, 42), while the rest of the studies performed internal validation by evaluating unseen portions of their training set. However, none of the studies conducted external validation using an independent and unseen set of data. This further implies that the prediction accuracy reported in these studies was based on a retrospective estimate rather than a prospective prediction and none of the studies tested their algorithms’ accuracy on future cases.

#### 3.3.4 Models results

Primary outcome measures for evaluating model performance included area under the receiver operating characteristic curve (AUROC), accuracy, sensitivity, specificity, positive predictive value (PPV), and negative predictive value (NPV), with AUROC and accuracy being the most frequently used performance metrics. Regarding each metric, the ranges, the proportion of studies reaching values ≥75%, and the best-performing study were as the following: AUROC (0.56 – 0.95; 15/17 studies reached ≥75% (38), reached the best value), sensitivity (8.33 – 95.23%, 11/14 studies reached ≥75% (38, 51), reached the best value), specificity (24.38 – 98.39%, 12/14 studies reached ≥75% (43), reached the best value), accuracy (50.27 – 90.67%, 12/15 studies reached ≥75% (38), reached the best value), PPV (14.58 – 94.45%, 6/10 studies reached ≥75% (45), reached the best value), NPV (20.48 – 99.34%, 8/10 studies reached ≥75% (39, 51), reached the best value). Additionally, twelve studies achieved values above 75% for both AUROC and accuracy.

#### 3.3.5 Models comparison

Running a meta-analysis on diverse studies with varying datasets, features, and variable distributions was impossible; Therefore, we adopted a particular approach to overcome the challenge of integrating and comparing the results of these studies. We specifically targeted studies that were designed to compare different models, as they offered valuable insights for our analysis. By extracting the rankings of different models, we could assess their relative performance, independent of the specific magnitude of each function indicator. This allowed us to overcome the limitations associated with diverse study designs and datasets, enabling a more meaningful comparison (**Table 2**).

**Table 2:** Performance ranks of each machine learning model across the different studies.

	ML Model	(37)	(39)	(45)	(43)	(41)	(46)	(49)	(50)	(48)	(47)	(51)	Mean
**Performance rank based on ACC**	GB	2.00	3.00	1.00	_	_	3.00	1.00	1.00	1.00	_	1.56	1.69
	NB	_	1.00	3.00	3.50	2.33	2.00	4.00	2.00	5.00	_	_	2.85
	RF	1.00	7.00	7.00	1.75	2.33	4.00	5.00	5.00	6.00	1.75	0.778	3.78
	DT	_	5.00	2.00	_	4.67	7.00	2.00	4.00	2.00	_	_	3.81
	LR	6.00	1.00	4.00	_	4.67	5.00	2.00	7.00	6.00	_	3.11	4.31
	KNN	_	6.00	5.00	0.88	1.17	6.00	6.00	6.00	3.00	_	6.22	4.47
	SVM	6.00	4.00	6.00	5.250	7.00	1.00	7.00	3.00	4.00	7.00	_	5.03
**Performance rank based on AUROC**	GB	5.00	1.00	1.00	_	_	2.00	1.00	2.00	1.00	_	0.78	1.72
	NB	_	2.00	2.00	6.13	1.17	2.00	3.00	1.00	3.00	_	_	2.54
	SVM	1.00	3.00	6.00	1.75	4.67	1.00	6.00	2.00	3.00	5.25	_	3.37
	RF	5.00	3.00	4.00	4.38	5.83	4.00	2.00	5.00	2.00	1.75	2.33	3.57
	LR	1.00	5.00	7.00	_	1.17	4.00	3.00	5.00	5.00	_	3.89	3.90
	KNN	_	6.00	2.00	7.00	1.17	6.00	7.00	4.00	6.00	_	7.00	5.13
	DT	_	7.00	5.00	_	7.00	7.00	3.00	7.00	7.00	_	_	6.14

ACC , Accuracy; AUROC , Area Under the Receiver Operator Characteristic Curve; GB , Gradient Boosting; NB , Naive Bayes; RF , Random Forest; DT , Decision Tree; LR , Logistic Regression; KNN , K-Nearest Neighbor; SVM , Support Vector Machine.

The ranks are within a range of 0 to 7. (For the studies that compared N models; all rankings were multiplied by 7/N.) A lower rank indicates a better prediction performance.

As mentioned earlier, thirteen studies were designed to compare different models (37, 39, 41–51). However, two of these studies utilized imaging data (38, 44), which differed from the data used in the other studies. Since each ML model typically performs well with specific types of data (65), combining the results of these two studies with the others was not appropriate. Therefore, we excluded these studies from the analysis to maintain standardization across the dataset, which left us with eleven studies.

The performance rank of each model across the different studies was aggregated to generate a final rank. This approach allowed us to understand the average success rate of each model. To enhance the interpretability of the results, we took two steps. Firstly, we excluded models used in less than half of the eleven studies. Secondly, we standardized the ranks so that they fell within a range of 0 to 7. (For the studies that compared N models, all rankings were multiplied by 7/N.) By doing so, we ensured that the final ranks accurately reflected the relative performance of each model. A lower final rank indicated a better average performance across the studies.

Finally, in terms of both accuracy and AUROC, the gradient boosting (GB) model consistently achieved the highest performance rank among the six models with a substantial margin compared to the next highest-ranked model. However, given that meta-analysis was not possible, it is not feasible to assess whether this margin was significant or not. This suggests that the GB model shows promising performance in predicting violence among SSD patients using clinical data.

### 3.4 Discriminative features

Various features were identified in the included studies as the predictor variables of VB in SSD patients. We can classify most of them into sociodemographic, clinical, metabolic, and neuroimaging groups. Most of the features were consistent in multiple studies, except for some discrepancies, which will be elaborated upon.

#### 3.4.1 Sociodemographic features

Some studies identified age (34, 37, 43, 45, 47), gender (34), and educational level (38, 43) as factors that contribute to the prediction of VB. However, other studies reported that these factors do not have a significant relationship with the occurrence of such behavior (35, 37, 44).

#### 3.4.2 Clinical features

Psychotic symptoms are associated with VB in SSD patients. Different studies consistently demonstrate that negative symptoms, such as flat affect and poverty of thought, decrease the risk of VB (35, 40). However, there is inconsistency in the results concerning the impact of positive symptoms on the occurrence of VB. Some studies suggest an increased risk of VB associated with positive symptoms (35, 43), while others propose a diminishing impact of specific positive symptoms, including delusion of persecution and auditory hallucination, on VB occurrence (40). Furthermore, various studies reported that daily dosage of prescribed olanzapine-equivalent at the time of discharge from previous psychiatric hospitalization of SSD patients can predict the occurrence of VB among them (39, 45, 46, 48, 49). However, their results were divergent, with four studies demonstrating a positive association between the olanzapine-equivalent dosage and risk of VB (39, 46, 48, 49), and one study reporting a negative association (45).

Patients’ past stresses also can contribute to VB. Patients who have experienced a higher number of past stressors had an increased risk of engaging in VB (42, 51). Consistently, history of previous outpatient psychiatric treatment was found to be associated with an increased risk of VB in patients (46, 48–50). In addition, specific stressors, including a history of coercive psychiatric treatment and separation from main caregivers in childhood or adolescence, have also been found to be related to VB (42). There is a lack of consensus on the relationship between patients’ employment status and VB. While Kirchebner et al. (2022) found a significant correlation between unemployment and VB (42), Chen et al. (2015) and Wang et al. (2020) reported no statistical relevance between a patient’s employment status and the likelihood of VB (35, 37).

Additionally, scores of several rating tools are significantly associated with VB. The BPRS total score, BPRS hostility score, BPRS withdrawal factors score (38), ITAQ score, family APGAR score, SSRS score, and FBS score (47) were all found to correlate with the risk of VB. Moreover, the PANSS total score at admission and discharge (39, 45), and PANSS anxiety and lack of spontaneity scores (50) are significantly related to VB. Other statistically relevant clinical features are presented in **Table 1**.

#### 3.4.3 Neuroimaging features

Two studies explored potential neuroimaging features for predicting VB. Gou et al. (2021) identified brain features associated with regional homogeneity (ReHo), gray matter volume (GMV), and fractional anisotropy (FA) as effective predictors of VB in schizophrenia patients (38). Significant GMV alterations were observed in the striatum system (including the putamen and pallidum), median cingulate, and paracingulate gyri, as well as temporal, occipital, and anterior parts of the parietal lobe. In addition, ReHo was most predictive in the anterior cingulate, dorsolateral part of the superior frontal gyrus, temporal pole, parietal lobe, and subcortical areas of the striatum, such as the caudate and pallidum. Also, the left superior longitudinal fasciculus was found to play a crucial role in FA predictions. Overall, the study identified the cingulate gyrus, dorsolateral part of superior frontal gyrus, temporal lobe (inferior temporal gyrus and temporal pole), supplementary motor area, and pallidum as the key regions for predicting VB in schizophrenia patients using sMRI and fMRI (38). On the other hand, Yu et al. (2022) found that the measurement of whole-brain GMV, right areas of superior temporal sulcus cortical thickness, right inferior parietal cortical thickness, and left frontal pole GMV correlated to the likelihood of violent tendencies (44).

#### 3.4.4 Metabolic features

Three plasma metabolites were recognized as potentially effective biomarkers for predicting VB. In the study by Chen et al. (2022) the ratio of L-asparagine to L-aspartic acid, vanillylmandelic acid, and glutaric acid was found to be associated with an increased likelihood of VB (36). Specifically, a decrease in the ratio of L-asparagine to L-aspartic acid and glutaric acid level and an increase in the vanillylmandelic acid level appear to be correlated with violent tendencies. Furthermore, altered specific metabolic pathways seemed to predispose individuals toward violence. Specifically, the glycerolipid metabolism pathway, characterized by an up-regulation of glycerol and a down-regulation of glycerol-3-phosphate, and the phenylalanine, tyrosine, and tryptophan biosynthesis pathway, marked by a down-regulation of 4-hydroxyphenylpyruvic, have been associated with violent tendencies (36). Moreover, it has been demonstrated that raised triglyceride levels were associated with a reduced likelihood of engaging in VB (35).

### 3.5 Risk of bias assessment

**Figure 2:**
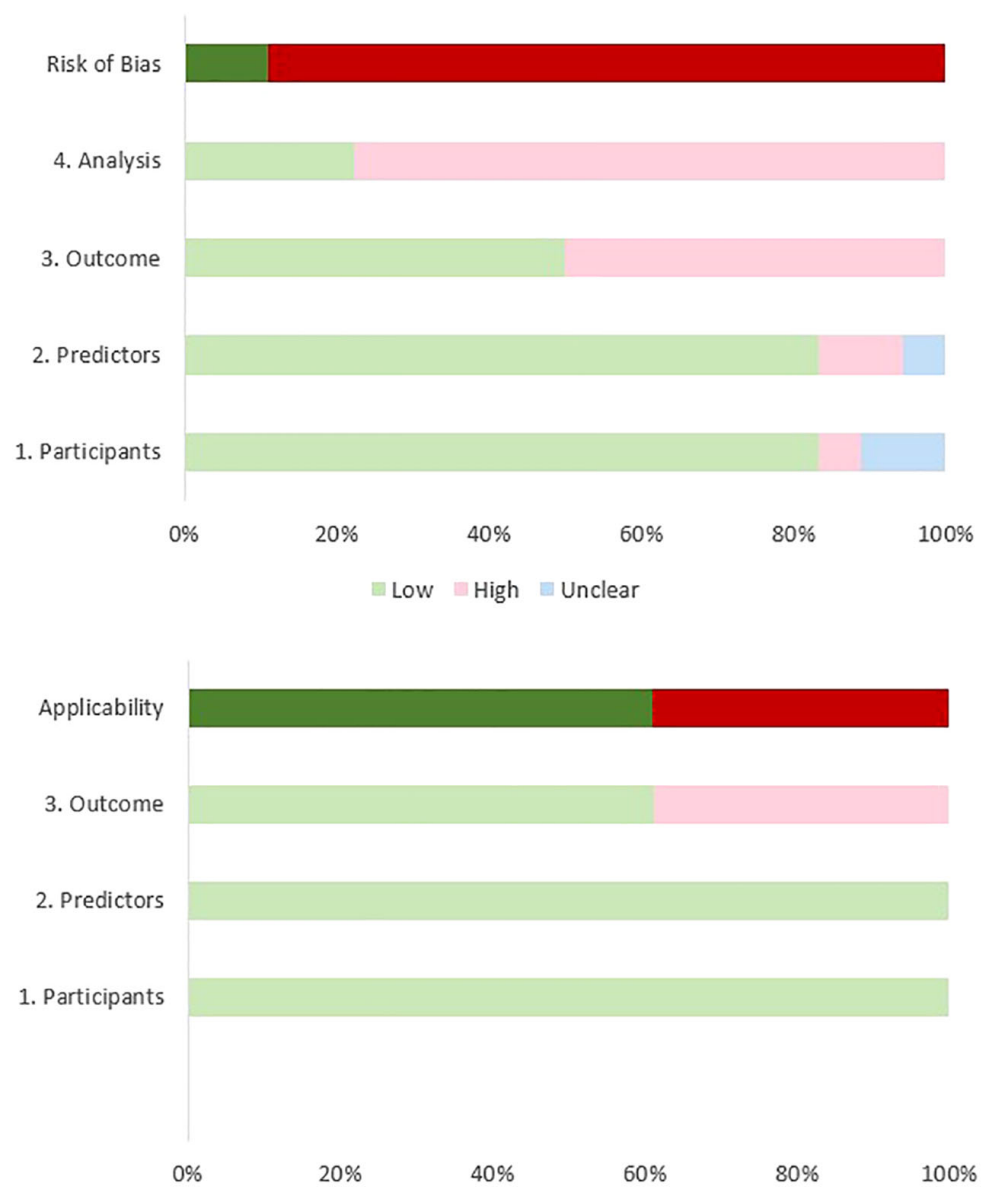
Assessment of Risk of Bias based on PROBAST.

Based on the results of our ROB assessment using the PROBAST guidelines, all studies except for two (43, 47), had some bias due to a small sample size, different violence definitions, and the inability to satisfy the study’s purpose. Although most of the studies had high ROB, the most important limitation arises from their limited sample sizes. According to the PROBAST guidelines, to achieve a low ROB in the analysis domain, the number of participants with the outcome relative to the number of the input variables should be equal to or higher than 20 (33). Only four reviewed studies had low ROB in the analysis domain (41, 42, 44, 47). Another reason for high ROB arises from the divergent definitions of violence and the use of different scales across the studies. We defined VB as an attempt or action to harm a target, assault, child or sexual abuse, and violent crimes. Whereas, Hofmann et al. (2022) included verbal aggression in the definition of VB (41) and four studies evaluated the ability of ML models to classify patients with previous criminal offenses (including VB) from non-offenders (46, 48–50). Also, some studies have evaluated the power of ML models in predicting VB (e.g., homicide) among offenders with SSD disorders (39, 42, 45). Although many studies represented high ROB in at least one field according to the PROBAST guideline (33), most of them (11/18) showed low concerns regarding applicability in the field of violence prediction in patients with SSD. **Table 3** and **Figure 2** illustrate the results of the quality assessment process.

**Table 3:** Findings of the ROB assessment based on the PROBAST statement.

Study	ROB				Applicability			Overall	
	Participants	Predictors	Outcome	Analysis	Participants	Predictors	Outcome	ROB	Applicability
(35)	–	+	–	–	+	+	+	–	+
(36)	+	+	+	–	+	+	+	–	+
(38)	+	–	+	–	+	+	+	–	+
(41)	+	?	–	+	+	+	–	–	–
(42)	?	–	–	+	+	+	+	–	+
(39)	+	+	–	–	+	+	–	–	–
(45)	+	+	–	–	+	+	–	–	–
(40)	?	+	+	–	+	+	+	–	+
(34)	+	+	+	–	+	+	+	–	+
(37)	+	+	+	–	+	+	+	–	+
(44)	+	+	+	–	+	+	+	–	+
(43)	+	+	+	+	+	+	+	+	+
(46)	+	+	–	–	+	+	–	–	–
(47)	+	+	+	+	+	+	+	+	+
(48)	+	+	–	–	+	+	–	–	–
(49)	+	+	–	–	+	+	–	–	–
(50)	+	+	–	–	+	+	–	–	–
(51)	+	+	+	–	+	+	+	–	+

PROBAST, Prediction Model Risk of Bias Assessment Tool; ROB, Risk of Bias.

+ indicates low ROB/low concerns regarding applicability; - indicates high ROB/high concerns regarding applicability; and? indicates unclear ROB/unclear concerns regarding applicability.

## 4 Discussion

### 4.1 Key findings

Previous research has shown an acceptable power for ML models in predicting VB in populations broader than SSD patients (68, 69). In this article, we reviewed the role of ML in predicting VB in patients with SSD. According to our findings, the predictive performances of the ML models varied across the reviewed papers. However, ML models performed better in studies that employed more intricate methodologies for model development and evaluation. These findings suggest that a well-designed ML model could be a potential tool for VB prediction in SSD patients, and could be beneficial in warning the caregivers to seek prevention techniques and stop them from further harmful acts in clinical and forensic settings. Among the reviewed ML models, GB showed the best performance in VB detection. Also, we reviewed the most discriminating features in violence prediction of SSD patients. Age (34, 37, 43, 45, 47) and olanzapine equivalent dose at the time of discharge (39, 45, 46, 48, 49) were the most repetitive variables found to be associated with violence across the studies.

### 4.2 Machine learning models

While direct comparison of results among studies was challenging due to the differences in sample characteristics, some insights were obtained. First, about two third of the studies (11/18) could reach values above 75% for both AUROC and accuracy, indicating that ML can be a promising tool for the accurate prediction of VB among SSD patients. Second, the studies demonstrated diverse performance in predicting VB among SSD patients, with an AUROC ranging from 0.56 to 0.95 and an accuracy range of 50.27% to 90.67%. However, the performance ranges within each study were narrower when comparing different ML models. Considering that many studies employed similar ML models and input variables, the observed diversity in performance appears to be partly influenced by the variations in study designs. This suggests that future similar studies could enhance their results not only by focusing on ML model selection or input variable choices but also by paying attention to the details of model development to mitigate biases.

In addition, there exists considerable divergence among the reviewed studies with regard to the methodologies employed for both feature selection and cross-validation. These two components play pivotal roles in the trajectory of ML model development, serving to mitigate overfitting and augment overall model performance (70). Within the included studies, a mere seven undertook data-driven feature selection utilizing ML techniques prior to model training as a preemptive measure against overfitting (38, 39, 41–43, 45). Notably, one study adopted a *post hoc* approach, selecting features subsequent to the computation of variable importance for each employed model independently (51). Additionally, sixteen studies embraced diverse methods for cross-validation, while two studies opted to forgo its application (35, 40). This heterogeneity in model development practices across the reviewed studies poses a significant obstacle to synthesizing their respective findings.

Therefore, our significant challenge was comparing ML models by integrating the results of different studies due to variations in sample characteristics, including differences in input and output variables distribution. To address this challenge, we devised a ranking method that enabled us to assess the overall success rate of commonly used methods. Based on our findings, the GB model exhibited notably superior average performance. However, it is essential to note that this does not necessarily imply inherent weakness in the other models. Instead, it highlights the favorable results achieved by the GB model in the specific context of the studied field.

GB is a subset of ensemble machine learning models, which also includes common models like classification trees and RF (32). This approach enables the effective handling of big data and also the handling of missing values in the predictors (32). While common ensemble techniques like RF rely on straightforward averaging of models within the ensemble, GB stands out for its step-by-step, sequential strategy for selecting the best predictor (71). This notable flexibility empowers GB to be highly adaptable to specific data-driven tasks (71). Due to its unique characteristics, GB outperformed other ML models in predicting VB in SSD patients, particularly due to its effective handling of a large number of predictors. Nevertheless, it is noteworthy that several other ML models, including SVM, LASSO, NNET, RF, decision trees, PDA, MLP, elastic net, and LR, in the studies reviewed, also achieved AUROC values exceeding 0.9 (41, 44, 47, 51). This highlights the substantial predictive potential of these alternative ML models in addition to GB.

### 4.3 Discriminative features

Notably, various studies have explored the influence of age on VB risk, yielding diverse findings. For instance, Tzeng et al. (2004) associated younger age with a higher risk of VB (34). In contrast, four other studies observed that older age correlated with an increased tendency for VB in SSD patients (37, 44, 45, 47). Yet, Chen et al. (2015) found no significant correlation between patients’ age and VB risk. While the majority of previous research aligns with Chen et al. (2015) and negates the association between age and VB risk (35, 72), there are outliers such as Soyka et al. (2007) who identified older ages as linked to a higher VB risk in SSD patients (73). This variability underscores the need for further research to ascertain the precise impact of age on VB occurrence in SSD patients.

Contrary to age, the reported gender effect on VB occurrence risk was quite consistent among studies, which showed that male sex was associated with a higher risk of VB (34). These findings confirmed most of the previous studies that reported a higher prevalence of VB among male SSD patients (73, 74), as the general population (17). Furthermore, Gou et al. (2021) and Yu et al. (2022), but not Chen et al. (2015) and Yu et al. (2022a), found that lower educational levels could predict VB occurrence (38, 43). This was in line with previous research that found lower educational levels to be significant predictors of VB among SSD patients (75, 76) and the general population (17). These disparities suggest that further studies on larger populations are required to determine the exact effect of the educational level of SSD patients on their VB tendency.

Moreover, in terms of the effect of occupational status on VB tendency, Kirchebner et al. (2022) reported a significant relationship between unemployment and VB in SSD patients (42), which confirmed the findings by Karabekiroğlu et al. (2016). Conversely, Chen et al. (2015) and Wang et al. (2020) found no correlation between employment status and VB (35, 37). This divergence could be a result of different definitions of violence in these studies; indeed, Kirchebner et al. (2022) and Karabekiroğlu et al. (2016) studies, unemployment was able to differentiate SSD patients with serious VB (e.g., homicide) from patients with minor VB (e.g., property damage). On the other hand, two other studies trained ML models to differentiate SSD patients with any kind of VB (serious or minor) from patients without VB (35, 37). This suggests that unemployment does not seem to be associated with the overall risk of VB among SSD patients, but it increases the risk of serious VB among offenders.

Regarding the clinical features, two studies reported that the presence of positive symptoms (35, 43) was correlated with an increased risk of VB, which was consistent with previous research (72, 74, 77). However, another study suggested that the presence of specific positive symptoms, including delusion of persecution and auditory hallucination, decreases the risk of VB (40). This controversy indicates that different types of delusion may have varying effects on the occurrence of VB (40). Also, Sonnweber et al. (2021, 2022) found a favorable predictive power for the PANSS total score of the patients in two different studies (39, 45). This is in line with previous studies that demonstrated higher PANSS total scores in violent patients, compared to non-violent patients (78, 79). Moreover, consistent with previous research (80), Gou et al. (2021) found that the risk of VB occurrence is higher among SSD patients with higher scores in the BPRS hostility subscale. Furthermore, higher scores in BPRS total score, BPRS withdrawal factors, PCL-SV, HCR-20 (38), and SDSS (43) successfully predicted VB in SSD patients across the reviewed studies. While the BPRS and PANSS scales assess various domains of SSD, including positive and negative symptoms (55, 81), the PCL-SV scale is specifically designed to evaluate psychopathic traits in patients, which is not directly associated with SSD (82). This indicates that aside from psychotic symptoms, additional symptoms like patients’ personality profiles, including psychopathy and impulsivity, may have relevance in predicting VB among individuals with SSD. Altogether, these suggest that by training ML models with certified psychiatric rating tools, we can significantly improve the accuracy of predicting VB in SSD patients, which can be highly beneficial in clinical applications.

Chen et al. (2015) found negative symptoms to be correlated with a decreased risk of VB (35), which is in line with previous studies that found depressive and other negative symptoms to be associated with a lower occurrence of VB in SSD patients (73, 77). Furthermore, the effect of the age of disease onset was controversial across the reviewed studies. While Sonnweber et al. (2021, 2022) reported that younger age of disease onset correlated with the probability of VB, Chen et al. (2015) and Wang et al. (2020) did not find a significant relationship between the age of disease onset and VB occurrence. The findings of previous research in this field are also divergent. Indeed, while Caqueo-Urízar et al. (2016) found VB to be more prevalent among patients with younger age of illness onset, Nolan et al. (1999) did not find any significant differences between the age of onset of violent and non-violent patients. Therefore, further research is warranted to determine the effects of disease onset on the VB occurrence in SSD patients, as it can help the early detection and treatment of patients at higher risk of VB.

While most studies evaluating the prescribed daily olanzapine-equivalent dose at the time of discharge from previous hospitalizations have reported a positive association with the risk of VB (39, 46, 48, 49), there is an exception in one study that reported the opposite (45). The divergence in findings can be attributed to the different focus of the Sonnweber (2022) study, which specifically differentiated between homicide committers and patients who committed other types of VB (45). It is logical to assume that higher doses of antipsychotics are prescribed to patients with more enduring symptoms, as they are reported to be more prone to engaging in VB in some studies (83). However, some previous studies found no significant association between the disease severity or prescribed dosage of antipsychotics and the risk of VB in patients with SSD (84, 85). This highlights the need for further research to better comprehend the relationship between disease severity and prescribed antipsychotic dosages in the occurrence of VB among SSD patients.

Previous research has shown that SSD patients’ previous history of violence is significantly correlated with increased risks of VB, such as recent violence episodes (86), history of a recent assault (87), previous history of aggression (74), and a previous violent conviction (87). Consistently, Sonnweber et al. (2021) reported previous conviction history as a significant predictor of VB in SSD patients (39). Moreover, Wang et al. (2020) found that a history of more than five times of hospitalization increased the likelihood of VB tendency in patients. However, Tzeng et al. (2004) reported that the lifetime number of hospitalizations was not correlated with an increased risk of VB occurrence in SSD patients. This disparity could be due to the differences in the psychiatric history assessment across the studies, as Tzeng et al. (2004) evaluated a broader variable (lifetime hospitalization), while other studies assessed the recent hospitalization history (88, 89) or a more distinguishing variable (≥ 5-lifetime hospital admissions) (37).

Finally, two studies have observed that neuroimaging variables were robust predictors of VB in SSD patients. Yu et al. (2022) found decreased whole-brain gray matter volume, right inferior parietal thickness, and left frontal pole volume to be predictors of VB. Consistently, Gou et al. (2021) reported disruption in the structural and functional MRI of the temporal, frontal lobes, cingulate gyrus, and striatum can predict VB in SSD patients. Also, a systematic review of 21 studies, revealed that reduced volumes of the frontal lobe in patients with schizophrenia are associated with a higher rate of VB occurrence (90). This is not surprising, as previous research mentioned a prominent role for frontal and temporal lobes and cingulate gyrus disruptions in developing VB (91). Considering the role of the frontal cortex in controlling disinhibited behaviors (e.g., impulsiveness, aggressiveness, and violence), patients with disrupted frontal cortex are more likely to present VB (91, 92). Although previous research established the involvement of the hippocampus and amygdala in emotional processing and in the development of VB (91), the predictive value of these regions was not assessed across the reviewed studies. In conclusion, our knowledge in the field of ML-based prediction of VB in SSD patients by training MRI data is still limited, and future research is required to clarify its potential.

### 4.4 Limitations and further directions

This study has some limitations. First, the sample sizes of most studies were small, considering the number of input variables, which can influence their analysis results. Second, the study samples across the reviewed articles were heterogeneous, as most of them studied clinical inpatients, while some studied forensic inpatients, and one included only outpatients’ data. Also, some studies only included male patients. Third, the outcome definitions differed within studies. For example, while most of the studies classified SSD patients into violent and non-violent, some others distinguished patients with serious types of VB (e.g., homicide) from other types of VB. Fourth, the reviewed studies were conducted in countries with different healthcare systems, which could have a significant impact on violence among SSD patients. Fifth, most of the studies did not select time-dependent features for VB prediction, which substantially lowers the ML model performance. Finally, none of the reviewed articles performed external validation, which can significantly diminish the generalizability of their findings. Therefore, future research with more homogenous methodologies and both internal and external validations seems to be necessary.

### 4.5 Conclusions

The outcomes of the ML models employed by the reviewed studies have yielded compelling findings, highlighting the significance of continuing along this research trajectory for further exploration and advancement. More in detail, while the ML models’ performance in VB prediction among SSD patients was divergent, yet promising, our comparative analysis demonstrated that GB outperformed other ML models. Considering the heterogeneity of ML model applications and study populations across the reviewed articles, there is substantial potential for further research in this field. Furthermore, the absence of external validation in the majority of the included articles reduces the generalizability of their findings. Indeed, subsequent research endeavors, employing comparable models, outcomes, and predictors, in extensive clinical samples, are imperative to substantiate the certainty of the current findings and ascertain the applicability of the developed ML algorithms.

Moreover, given the rapidly growing trend in the application of various artificial intelligence tools in medical contexts, it appears likely that in the next years ML models can be also utilized for VB prediction in SSD patients. Indeed, while the performance of ML models varied across the reviewed studies; several models demonstrated excellent predictive abilities with an AUROC exceeding 0.9. This highlights the potential for developing reliable ML models through further well-designed studies. Upon validation through external assessments, these models could effectively predict VB in real-world clinical settings. Consequently, the development of clinical assessment tools integrating patient data could facilitate the early identification of individuals highly susceptible to VB, whether in outpatient or inpatient settings. The utilization of such tools enables timely preventive interventions, such as providing social support and rehabilitation, adjusting medications, and considering more intensive therapeutic approaches, like electroconvulsive therapy. Implementing these measures could significantly alleviate the burden of VB on patients, healthcare systems, and society at large.

## Data availability statement

The original contributions presented in the study are included in the article/**Supplementary Material**. Further inquiries can be directed to the corresponding authors.

## Author contributions

MP: Conceptualization, Investigation, Project administration, Resources, Visualization, Writing – original draft. AA: Data curation, Investigation, Visualization, Writing – original draft. MT: Data curation, Formal Analysis, Writing – original draft. HS: Writing – original draft. GC: Funding acquisition, Supervision, Writing – review & editing. FS: Supervision, Writing – review & editing. AP: Data curation, Writing – review & editing. PB: Project administration, Supervision, Writing – review & editing. GD: Funding acquisition, Methodology, Supervision, Writing – review & editing.

## Glossary

**Table:**

AIS	Aggressive Incidents Scale
APGAR	Family Adaptation, Partnership, Growth, Affection and Resolve
AUROC	area under the receiver operating characteristic curves
BIS-11	Barratt Impulsiveness Scale version 11
BPRS	Brief Psychiatric Rating Scale
DSM	Diagnostic and Statistical Manual of Mental Disorders
DTI	diffusion tensor imaging
FA	fractional anisotropy
FBS	Family Burden Scale of Disease
fMRI	Functional magnetic resonance imaging
GB	gradient boosting
GMV	gray matter volume
HCR-20	Historical, Clinical and Risk management
ICD	International Classification of Diseases
ITAQ	Insight and Treatment Attitude Questionnaire
KNN	k-nearest neighbor
LASSO	least absolute shrinkage and selection operator
LR	logistic regression
ML	machine learning
MOAS	Modified Overt Aggression Scale
MPL	multi-layer perceptron
MRI	magnetic resonance imaging
MVRAS	MacArthur Violence Risk Assessment Study
NNET	neural network
NPV	negative predictive values
PANSS	Positive And Negative Symptom Scale
PCL-SV	Psychopathy Checklist, Screening Version
PPV	positive predictive values
PRISMA	Preferred Reporting Items for Systematic Reviews and Meta-Analysis
PROBAST	Prediction Model Risk of Bias Assessment Tool
ReHo	regional homogeneity
RF	random forests
ROB	risk of bias
SDSS	Social Disability Screening Schedule
sMRI	structural magnetic resonance imaging
SSD	schizophrenia spectrum disorders
SSRS	Social Support Rating Scale
SVM	support vector machine
VAS-A	Violence and Suicide Assessment
VB	violent behaviors
