# Supplementary material for: Machine Learning for prediction of violent behaviors in schizophrenia spectrum disorders: a systematic review
Source: Front Psychiatry. 2024 Mar 21;15:1384828. doi: 10.3389/fpsyt.2024.1384828 (PMC10991827; doi:10.3389/fpsyt.2024.1384828)
Supplement: Supplementary file 1 [file DataSheet_1.docx]

Supplementary Material

# Supplementary Data

**1.1 Search Terms (September 30, 2023):**

(“Schizophrenia” OR “Schizophrenia Spectrum Disorder” OR “Schizophrenic” OR “Schizoaffective” OR “Schizophreniform” OR “Delusional Disorder” OR “Psychotic Disorder Not Otherwise Specified” OR “Psychotic Disorder NOS”) AND ("Viol*” OR "Offens*" OR “Aggressive” OR “Aggression” OR “Aggressor” OR “Impuls*” OR “Hostil*” OR “Anger*” OR “Bull” OR “Bully” OR “Bullying” OR ((“External” OR “Problem” OR “Maladaptive”) AND (“Behavio*”)) OR “Anti-social” OR “Antisocial” OR "Sadis*” OR “Dangerous” OR “Criminal” OR "Crime" OR “Legal” OR “Forensic” OR "Cruel*" OR "Homicid*” OR "Murder" OR "Murderer" OR "Killing" OR "Assassin*" OR "Assault" OR "Brutal*" OR “Intimidation”) AND ("Machine Learning" OR “Deep Learning” OR “Algorithm” OR “Algorithms” OR “Iterative Learning” OR "Supervised Learning" OR "Unsupervised Learning" OR "Semi-Supervised Learning" OR “Reinforced Learning” OR "Artificial Intelligence" OR "Support Vector Machine" OR "Random Forest" OR "Linear Regression" OR "Logistic Regression" OR "Decision Tree" OR "Gradient Boosting" OR “Gaussian Naïve Bayes” OR “Gaussian Naive Bayes” OR “Extreme Gradient Boosting” OR “Decision Tree Classifier” OR “Linear Discriminant Analysis” OR “K-Nearest Neighbor” OR “K-Means” OR “Multilayer Perception” OR “Least Absolute Shrinkage and Selection Operator” OR "LASSO" OR “Iterative Self-Organizing Data Analysis Technique” OR "ISODATA" OR “Primary Component Analysis” OR “Principal Component Analysis” OR “Independent Component Analysis”)

**1.2 Searched Databases:**

**1. PUBMED:** search keywords were conducted through “All Fields”, with no filters.

**2. Embase:** search keywords were conducted through “Broad Search”, and the “Article” filter was applied.

**3. Web of Science:** search keywords were conducted through “All Fields”, and the “Article” filter was applied.

**4. PsycINFO:** search keywords were conducted through “All Text”, and the “Peer-Reviewed Journal” filter was applied.

# PROBAST Domains

**Domain 1:** **Participants**

***Risk of bias assessment***

Background: The overall aim for prediction models is to generate absolute risk predictions that are correct in new individuals. Certain data sources or designs are not suited to generate absolute probabilities. Problems may also arise if a study inappropriately includes or excludes participant groups from entering the study.

1.1 Were appropriate data sources used, e.g., cohort, RCT, or nested case–control study data?

Yes/probably yes: If a cohort design (including RCT or proper registry data) or a nested case–control or case–cohort design (with proper adjustment of the baseline risk/hazard in the analysis) has been used.

No/probably no: If a nonnested case–control design has been used.

No information: If the method of participant sampling is unclear.

1.2 Were all inclusions and exclusions of participants appropriate?

Yes/probably yes: If inclusion and exclusion of participants was appropriate, so participants correspond to unselected participants of interest.

No/probably no: If participants are included who would already have been identified as having the outcome and so are no longer participants at suspicion of disease (diagnostic studies) or at risk of developing outcome (prognostic studies), or if specific subgroups are excluded that may have altered the performance of the prediction model for the intended target population.

No information: When there is no information on whether inappropriate inclusions or exclusions took place.

***** Risk of bias introduced by participants or data sources:

Low risk of bias: If the answer to all signaling questions is “Yes” or “Probably yes,” then risk of bias can be considered low. If ≥1 of the answers is “No” or “Probably no,” the judgment could still be “Low risk of bias” but specific reasons should be provided why the risk of bias can be considered low.

High risk of bias: If the answer to any of the signaling questions is “No” or “Probably no,” there is a potential for bias, except if defined at low risk of bias above.

Unclear risk of bias: If relevant information is missing for some of the signaling questions and none of the signaling questions is judged to put this domain at high risk of bias.

***Applicability***

Background: Included participants, the selection criteria used as well as the setting used in the primary prediction model study should be relevant to the review question. Concern that included participants or the setting do not match the review question.

Low concern for applicability: Included participants and clinical setting match the review question.

High concern for applicability: Included participants and clinical setting were different from the review question.

Unclear concern for applicability: If relevant information.

**Domain 2:** **Predictors**

***Risk of bias assessment***

Background: Bias in model performance can occur when the definition and measurement of predictors is flawed. Predictors are the variables evaluated for their association with the outcome of interest. Bias can occur, for example, when predictors are not defined in a similar way for all participants or knowledge of the outcome influences predictor assessments.

2.1 Were predictors defined and assessed in a similar way for all participants?

Yes/probably yes: If definitions of predictors and their assessment were similar for all participants.

No/probably no: If different definitions were used for the same predictor or if predictors requiring subjective interpretation were assessed by differently experienced assessors.

No information: If there is no information on how predictors were defined or assessed.

2.2 Were predictor assessments made without knowledge of outcome data?

Yes/probably yes: If outcome information was stated as not used during predictor assessment or was clearly not (yet) available to those assessing predictors.

No/probably no: If it is clear that outcome information was used when assessing predictors.

No information: No information on whether predictors were assessed without knowledge of outcome information.

2.3 Are all predictors available at the time the model is intended to be used?

Yes/probably yes: All included predictors would be available at the time the model is intended to be used for prediction.

No/probably no: Predictors would not be available at the time the model is intended to be used for prediction.

No information: No information on whether predictors would be available at the time the model is intended to be used for prediction.

* Risk of bias introduced by predictors or their assessment:

Low risk of bias: If the answer to all signaling questions is “Yes” or “Probably Yes,” then risk of bias can be considered low.

If ≥1 of the answers is “No” or “Probably no,” the judgment could still be “Low risk of bias” but specific reasons should be provided why the risk of bias can be considered low, e.g., use of objective predictors not requiring subjective interpretation.

High risk of bias: If the answer to any of the signaling questions is “No” or “Probably no,” there is a potential for bias.

Unclear risk of bias: If relevant information is missing for some of the signaling questions and none of the signaling questions is judged to put the domain at high risk of bias.

***Applicability***

Background: The definition, assessment, and timing of predictors in the primary prediction model study should be relevant to the review question, e.g., predictors should be measured using methods potentially applicable to the daily practice that is addressed by the review. Concern that the definition, assessment, or timing of predictors in the model do not match the review question.

Low concern for applicability: Definition, assessment, and timing of predictors match the review question.

High concern for applicability: Definition, assessment, or timing of predictors were different from the review question.

Unclear concern for applicability: If relevant information about the predictors is not reported.

**Domain 3:** **Outcome**

***Risk of bias assessment***

Background: Bias in model performance can occur when methods used to determine outcomes incorrectly classify participants with or without the outcome. Bias in methods of outcome determination can result from use of suboptimal methods, tests, or criteria that lead to unacceptably high levels of errors in outcome determination, when methods are inconsistently applied across participants, or when knowledge of predictors influence outcome determination. Incorrect timing of outcome determination can also result in bias.

3.1 Was the outcome determined appropriately?

Yes/probably yes: If a method of outcome determination has been used which is considered optimal or acceptable by guidelines or previous publications on the topic.

Note: This is about level of measurement error within the method of determining the outcome (see concerns for applicability about whether the definition of the outcome method is appropriate).

No/probably no: If a clearly suboptimal method has been used that causes unacceptable error in determining outcome status in participants.

No information: No information on how outcome was determined.

3.2 Was a prespecified or standard outcome definition used?

Yes/probably yes: If the method of outcome determination is objective, or if a standard outcome definition is used, or if prespecified categories are used to group outcomes.

No/probably no: If the outcome definition was not standard and not prespecified.

No information: No information on whether the outcome definition was prespecified or standard.

3.3 Were predictors excluded from the outcome definition?

Yes/probably yes: If none of the predictors are included in the outcome definition.

No/probably no: If ≥1 of the predictors forms part of the outcome definition.

No information: No information on whether predictors are excluded from the outcome definition.

3.4 Was the outcome defined and determined in a similar way for all participants?

Yes/probably yes: If outcomes were defined and determined in a similar way for all participants.

No/probably no: If outcomes were clearly defined and determined in a different way for some participants.

No information: No information on whether outcomes were defined or determined in a similar way for all participants.

3.5 Was the outcome determined without knowledge of predictor information?

Yes/probably yes: If predictor information was not known when determining the outcome status, or outcome status determination is clearly reported as determined without knowledge of predictor information.

No/probably no: If it is clear that predictor information was used when determining the outcome status.

No information: No information on whether outcome was determined without knowledge of predictor information.

3.6 Was the time interval between predictor assessment and outcome determination appropriate?

Yes/probably yes: If the time interval between predictor assessment and outcome determination was appropriate to enable the correct type and representative number of relevant outcomes to be recorded, or if no information on the time interval is required to allow a representative number of the relevant outcome occur or if predictor assessment and outcome determination were from information taken within an appropriate time interval.

No/probably no: If the time interval between predictor assessment and outcome determination is too short or too long to enable the correct type and representative number of relevant outcomes to be recorded.

No information: If no information was provided on the time interval between predictor assessment and outcome determination.

* Risk of bias introduced by predictors or their assessment:

Low risk of bias: If the answer to all signaling questions is “Yes” or “Probably yes,” then risk of bias can be considered low. If ≥1 of the answers is “No” or “Probably no,” the judgment could still be low risk of bias, but specific reasons should be provided why the risk of bias can be considered low, e.g., when the outcome was determined with knowledge of predictor information but the outcome assessment did not require much interpretation by the assessor (e.g., death regardless of cause).

High risk of bias: If the answer to any of the signaling questions is “No” or “Probably no,” there is a potential for bias. Unclear risk of bias: If relevant information about the outcome is missing for some of the signaling questions and none of the signaling questions is judged to put this domain at high risk of bias.

***Applicability***

Background: The definition of outcome in the primary study should be relevant for the outcome definition in the review question. Concern that the outcome definition, timing, or determination do not match the review question

Low concern for applicability: Outcome definition, timing, and method of determination defines the outcome as intended by the review question.

High concern for applicability: Choice of outcome definition, timing, and method of outcome determination defines another outcome as intended by the review question.

Unclear concern for applicability: If relevant information about the outcome, timing, and method of determination is not reported.

**Domain 4:** **Analysis**

***Risk of bias assessment***

Background: Statistical analysis is a critical part of prediction model development and validation. The use of inappropriate statistical analysis methods increases the potential for bias in reported model performance measures. Model development studies include many steps where flawed methods can distort results. We recommend reviewers seek statistical advice when completing assessments of the analysis domain.

4.1 Were there a reasonable number of participants with the outcome?

Yes/probably yes: For model development studies, if the number of participants with the outcome relative to the number of candidate predictor parameters is ≥20 (EPV ≥20).* For model validation studies, if the number of participants with the outcome is ≥100.

No/probably no: For model development studies, if the number of participants with the outcome relative to the number of candidate predictor parameters is <10 (EPV <10).* For model validation studies, if the number of participants with the outcome is <100.

No information: For model development studies, no information on the number of candidate predictor parameters or number of participants with the outcome, such that the EPV cannot be calculated. For model validation studies, no information on the number of participants with the outcome.

4.2 Were continuous and categorical predictors handled appropriately?

Yes/probably yes: If continuous predictors are not converted into ≥2 categories when included in the model (i.e., dichotomized or categorized), or if continuous predictors are examined for nonlinearity using, for example, fractional polynomials or restricted cubic splines, or if categorical predictor groups are defined using a prespecified method. For model validation studies, if continuous predictors are included using the same definitions or transformations, and categorical variables are categorized using the same cut points, as compared with the development study.

No/probably no: If categorical predictor group definitions do not use a prespecified method.

For model development studies, if continuous predictors are converted into ≥2 categories when included in the model. For model validation studies, if continuous predictors are included using different definitions or transformations, or categorical variables are categorized using different cut points, as compared with the development study.

No information: No information on whether continuous predictors are examined for nonlinearity and no information on how categorical predictor groups are defined. For model validation studies, no information on whether the same definitions or transformations and the same cut points are used, as compared with the development study.

4.3 Were all enrolled participants included in the analysis?

Yes/probably yes: If all participants enrolled in the study are included in the data analysis.

No/probably no: If some or a subgroup of participants are inappropriately excluded from the analysis.

No information: No information on whether all enrolled participants are included in the analysis.

4.4 Were participants with missing data handled appropriately?

Yes/probably yes: If there are no missing values of predictors or outcomes and the study explicitly reports that participants are not excluded on the basis of missing data, or if missing values are handled using multiple imputation.

No/probably no: If participants with missing data are omitted from the analysis, or if the method of handling missing data is clearly flawed, e.g., missing

indicator method or inappropriate use of last value carried forward,

or if the study had no explicit mention of methods to handle missing data.

No information: If there is insufficient information to determine if the method of handling missing data is appropriate.

4.5 Was selection of predictors based on univariable analysis avoided?†

Yes/probably yes: If the predictors are not selected on the basis of univariable analysis prior to multivariable modeling.

No/probably no: If the predictors are selected on the basis of univariable analysis prior to multivariable modeling.

No information: If there is no information to indicate that univariable selection is avoided.

4.6 Were complexities in the data (e.g., censoring, competing risks, sampling of control participants) accounted for appropriately?

Yes/probably yes: If any complexities in the data are accounted for appropriately, or if it is clear that any potential data complexities have been identified appropriately as unimportant.

No/probably no: If complexities in the data that could affect model performance are ignored.

No information: No information is provided on whether complexities in the data are present or accounted for appropriately if present.

4.7 Were relevant model performance measures evaluated appropriately?

Yes/probably yes: If both calibration and discrimination are evaluated appropriately (including relevant measures tailored for models predicting survival outcomes).

No/probably no: If both calibration and discrimination are not evaluated, or if only goodness-of-fit tests, such as the Hosmer–Lemeshow test, are used to evaluate calibration, or if for models predicting survival outcomes performance measures accounting for censoring are not used, or if classification measures (like sensitivity, specificity, or predictive values) were presented using predicted probability thresholds derived from the data set at hand.

No information: Either calibration or discrimination are not reported, or no information is provided as to whether appropriate performance measures for survival outcomes are used (e.g., references to relevant literature or specific mention of methods, such as using Kaplan–Meier estimates), or no information on thresholds for estimating classification measures is given.

4.8 Were model overfitting and optimism in model performance accounted for?†

Yes/probably yes: If internal validation techniques, such as bootstrapping and cross-validation including all model development procedures, have been used to account for any optimism in model fitting, and subsequent adjustment of the model performance estimates have been applied.

No/probably no: If no internal validation has been performed, or if internal validation consists only of a single random split-sample of participant data, or if the bootstrapping or cross-validation did not include all model development procedures including any variable selection.

No information: No information is provided on whether internal validation techniques, including all model development procedures, have been applied.

4.9 Do predictors and their assigned weights in the final model correspond to the results from the reported multivariable analysis?†

Yes/probably yes: If the predictors and regression coefficients in the final model correspond to reported results from multivariable analysis.

No/probably no: If the predictors and regression coefficients in the final model do not correspond to reported results from multivariable analysis.

No information: If it is unclear whether the regression coefficients in the final model correspond to reported results from multivariable analysis.

* Risk of bias introduced by the analysis:

Low risk of bias: If the answer to all signaling questions is “Yes” or “Probably yes,” then risk of bias can be considered low. If ≥1 of the answers is “No” or “Probably no,” the judgment could still be low risk of bias, but specific reasons should be provided why the risk of bias can be considered low.

High risk of bias: If the answer to any of the signaling questions is “No” or “Probably no,” there is a potential for bias.

Unclear risk of bias: If relevant information about the analysis is missing for some of the signaling questions but none of the signaling question answers is judged to put the analysis at high risk of bias.

**RCT:** randomized controlled trial, **EPV:** events per variable.

* For EPVs between 10 and 20, the item should be rated as either probably yes or probably no, depending on the outcome frequency, overall model performance, and distribution of the predictors in the model. For more guidance, see references 145 to 147.

† Development only.
